# Embryonic development and secondary axis induction in the Brazilian white knee tarantula *Acanthoscurria geniculata*, C. L. Koch, 1841 (Araneae; Mygalomorphae; Theraphosidae)

**DOI:** 10.1007/s00427-020-00653-w

**Published:** 2020-02-19

**Authors:** Matthias Pechmann

**Affiliations:** Institute for Zoology, Department for Developmental Biology, Biocenter, University of Cologne, Zuelpicher Str. 47b, 50674 Cologne, Germany

**Keywords:** Mygalomorphae, *Acanthoscurria geniculata*, Embryonic development, Axis duplication, Cumulus grafting, Bead transplantation

## Abstract

Tarantulas represent some of the heaviest and most famous spiders. However, there is little information about the embryonic development of these spiders or their relatives (infraorder Mygalomorphae) and time-lapse recording of the embryonic development is entirely missing. I here describe the complete development of the Brazilian white knee tarantula, *Acanthoscurria geniculata,* in fixed and live embryos. The establishment of the blastoderm, the formation, migration and signalling of the cumulus and the shape changes that occur in the segment addition zone are analysed in detail. In addition, I show that there might be differences in the contraction process of early embryos of different theraphosid spider species. A new embryonic reference transcriptome was generated for this study and was used to clone and analyse the expression of several important developmental genes. Finally, I show that embryos of *A. geniculata* are amenable to tissue transplantation and bead insertion experiments. Using these functional approaches, I induced axis duplication in embryos via cumulus transplantation and ectopic activation of BMP signalling. Overall, the mygalomorph spider *A. geniculata* is a useful laboratory system to analyse evolutionary developmental questions, and the availability of such a system will help understanding conserved and divergent aspects of spider/chelicerate development.

**Electronic supplementary material:**

The online version of this article (10.1007/s00427-020-00653-w) contains supplementary material, which is available to authorized users.

## Introduction

Within the chelicerates, the spiders have become an important model system to study evolutionary, developmental and ecological questions. Furthermore, the diverse field of applications for spider venom and silk have always attracted scientists to study these fascinating animals. Several spider genomes have been published recently allowing to perform functional and evolutionary genomic studies (Kono et al. 2019; Sánchez-Herrero et al. 2019; Sanggaard et al. 2014; Schwager et al. 2017).

**Fig. 1:**
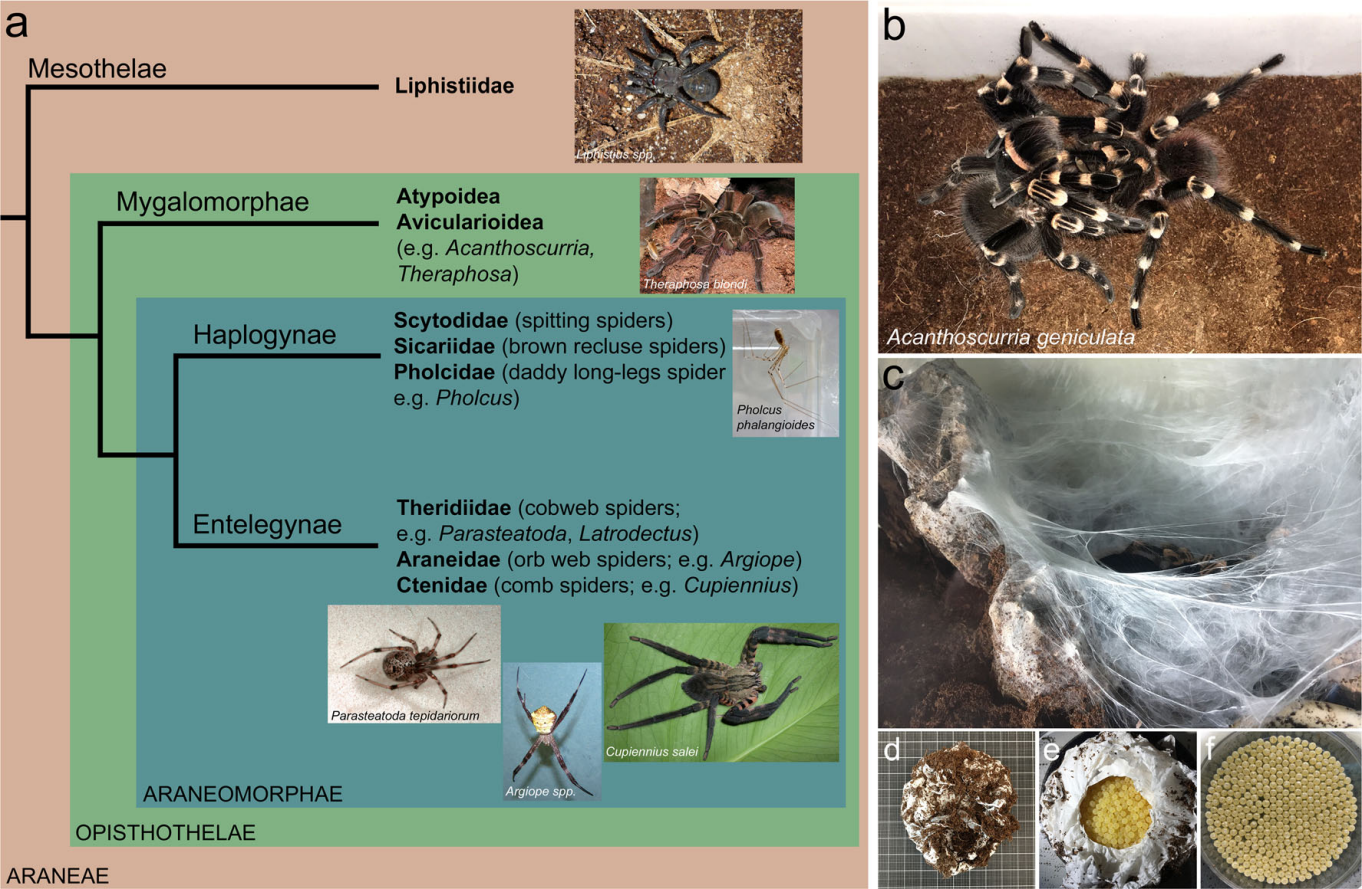
*A. geniculata* and relatives. **a** Spider phylogeny (simplified after Garrison et al. 2016) with famous representatives of each big branch. Embryonic development has been described in detail for *P. phalangioides* (Turetzek and Prpic 2016), *P. tepidariorum* (Mittmann and Wolff 2012) and *C. salei* (Wolff and Hilbrant 2011). **b** Mating pair of *A. geniculata*. **c** Female of *A. geniculata* during cocoon production. **d** A cocoon of *A. geniculata* on scaling paper. **e** Opened cocoon. **f** 370 eggs (around a fifth of eggs of the cocoon shown in **d** and **e**) in a petri dish of 5.5 cm diameter

Spiders (*Araneae*; around 48000 described species (World Spider Catalog 2019)) can be divided into three suborders; the basally branching segmented Mesothelae, the Mygalomorphae (tarantulas, trapdoor spiders and allies) and the Araneomorphae (“true spiders” including Haplogynae and Entelegynae) (see Fig. 1a).

In recent years, embryonic development has been extensively studied in Araneomorphae (e.g., Akiyama-Oda and Oda 2003; Mittmann and Wolff 2012; Turetzek and Prpic 2016; Wolff and Hilbrant 2011; Yamazaki et al. 2005). Although several classical, and also some recent, studies deal with the embryonic development of mygalomorph spiders and even Mesothelae (e.g., Crome 1963; Pechmann and Prpic 2009; Setton et al. 2019; Yoshikura 1954; Yoshikura 1958), fewer details on the embryonic development are available for these spider suborders. Furthermore, time-lapse recording of the embryonic development has proved incredibly useful for analysing araneomorphs (e.g., Mittmann and Wolff 2012; Turetzek and Prpic 2016; Wolff and Hilbrant 2011), but this approach has never been applied to other groups.

There are some important morphological differences between mygalomorph and araneomorph spiders which make it interesting to analyse and compare the development of these morphological characters in these two suborders of spiders. The first major morphological difference is the arrangement of the chelicerae. Until recently, this feature was used to place Mygalomorphae and Araneomorphae into the groups Orthognatha (chelicerae run in parallel to each other) and Labidognatha (chelicerae are in a vertical position opposing each other) (reviewed in e.g., Foelix 2011). As the chelicerae of Mesothelae also run in parallel to each other, it is likely to be the ancestral character of spiders. Other important morphological differences are the absence of the anterior pair of spinnerets in mygalomorph spiders (reviewed in e.g., Foelix 2011, Pechmann et al. 2010) and the presence of two pairs of book lungs in Mygalomorphae and Mesothelae vs. only one pair of book lungs in most Araneomorphae (exception Hypochilidae (e.g., Sharma 2017)). First studies on the embryonic development of tarantulas revealed, that the anterior spinnerets are present as rudimentary structures during the early embryonic development (e.g., Pechmann and Prpic 2009; Setton et al. 2019). It will be interesting to analyse the factors that are responsible for the loss or for the sustainment of the spinnerets during embryonic development. Establishment of a mygalomorph spider model system in the laboratory is thus crucial for the understanding of the evolution and the development of spiders.

Although mygalomorph spider species have a very long generation cycle (most species become sexual reproductive after several years), a large spider culture and the huge amount of eggs per cocoon can at least partially compensate for the long generation time of these animals. In addition, mygalomorph spiders produce very large eggs, which make them amenable to perform classical transplantation experiments (this study).

Here, I provide a detailed analysis of the embryonic development of the tarantula *A. geniculata*. In addition, I show that embryos of *A. geniculata* can be used to perform tissue grafting as well as bead insertion experiments. Applying these techniques, I show that the grafting of the “cumulus” (the organizing centre of early spider embryos) as well as the local activation of the BMP signalling pathway can induce an axis duplication phenotype.

Overall, *A. geniculata* is a promising system that can be used to perform modern and classical embryological studies. The availability of a draft genome assembly (Sanggaard et al. 2014), the presence of transcriptomic data (this work and Sanggaard et al. 2014) as well as the accessibility to modern molecular biological techniques make this system attractive to analyse a variety of biological questions.

## Methods

### Spider husbandry

All spiders used for this study originated from a laboratory culture (see Pechmann and Prpic 2009) or were purchased from German breeders. Wild caught animals have not been used. Juveniles of *A. geniculata* and *Brachypelma albopilosum* were kept in *Drosophila* vials and plastic boxes of appropriate size. Adults were kept in glass containers of 20 cm height, 30 cm width and 30 cm depth. Spiders were kept on moist coconut fibres and were fed with *Drosophila melanogaster* and *Gryllus bimaculatus* once or twice a week. Adults had access to a water dish. To speed up the development, juveniles were kept in incubators at 27 **°**C with 10 h light and 14 h dark cycle. Adults were kept in the lab at room temperature (near the window; without direct sun light). As the temperature dropped below 20 **°**C during the winter, an external heating source (thermo mat, 7 W, Lucky Reptile) was attached to one side of the glass container. Prior to and after mating, adult females were fed more frequently. Cocoons were opened with fine scissors, and eggs were kept within the opened cocoon or were transferred to petri dishes. Cocoons and eggs were incubated in large petri dishes containing a wet paper towel to ensure the desired humidity.

Worldwide, many hobbyists keep mygalomorph spiders as a pet. For this reason, several excellent books are available that describe the breeding and the maintenance of tarantulas in captivity (e.g., Klaas 2013; Schultz and Schultz 2009).

### Transcriptome sequencing and assembly

Total RNA from st. 5–14 embryos was extracted using TRIZOL and the Quick-RNA MicroPrep Kit (Zymo Research). In addition total RNA was extracted from fixed st. 5 embryos using the Quick-RNA FFPE Kit (Zymo Research). Both samples were sequenced individually.

Library preparation and sequencing was carried out at the Cologne Center for Genomics (Illumina HiSeq4000). The sequenced reads were subjected to adaptor and quality threshold trimming using Trimmomatic 0.33 (Bolger et al. 2014; Trimmomatic 0.33 settings: ILLUMINACLIP:adapter.fa:2:30:10 LEADING:3 TRAILING:3 SLIDINGWINDOW:4:15 MINLEN:30 where “adapter.fa” contained the adapters used in sequencing). FastQC (Andrews 2010) was used to assess read quality both before and after trimming. There was no difference in sequence quality between the two sequenced samples. The final transcriptome was assembled from both read sets using Trinity 2.6.6 (Grabherr et al. 2011; Trinity 2.6.6 settings: all default options, normalized reads, --CPU 8 --full_cleanup --inchworm_cpu 8 --bflyHeapSpaceMax 15G –bflyCalculateCPU), and the combined assembly can be found on Figshare (10.6084/m9.figshare.10266314). BioProject accession for the raw reads is PRJNA588224.

The statistics (see Table S2) were calculated using BUSCO v2/3 (Simão et al. 2015), the gVolante server (https://gvolante.riken.jp/analysis.html; Nishimura et al. 2017) and custom scripts that are available upon request.

### Gene cloning

CLC Main Workbench 7 (QIAGEN Aarhus A/S) was used to perform a local TBLASTN (Altschul et al. 1990) against the newly assembled transcriptome. For this, protein sequences of known homology were downloaded from FlyBase (Thurmond et al. 2019) and NCBI. Putatively identified genes were reciprocally blasted against the online NCBI nr database using BLASTx to confirm their identity.

cDNA synthesis was carried out using the SuperScript VILO cDNA Synthesis Kit (Thermo Fisher Scientific) or the qScript cDNA Synthesis Kit (Quanta bio). Total RNA or cDNA is available upon request.

PCR amplification and cloning of genes were performed using standard techniques. All genes were cloned into pCRII-TOPO or pCR4-TOPO (Invitrogen). A primer list with corresponding TRINITY_IDs of the genes found in the new embryonic reference transcriptome is provided in the supplemental material (Table S1).

### Imaging and image analysis

Most images were recorded using an AxioZoom.V16 (Zeiss; ZEN2 Software), equipped with a movable stage. For live imaging, the membrane of a cell culture dish (lumox dish 35, SARSTEDT) was covered with heptane glue. After the heptane evaporated, the embryos were glued to the membrane and covered with oil (Voltalef H10S or Halocarbon oil 700; SIGMA). Live imaging was performed at room temperature (25 **°**C ± 2 **°**C)**.**

The embryos shown in Movie S1, S2, S3, S6 and S9 were imaged via a 45-degree mirror as described in Pechmann 2016.

Helicon Focus (Helicon Soft) was used to generate image stack projections. Fiji (Schindelin et al. 2012) was used to create movies. Created movies were converted and compressed using HandBrake (https://handbrake.fr/). Adobe Photoshop CS5.1 was used to adjust for brightness and contrast.

False color overlays of in situ hybridisation images were generated as described in Pechmann et al. 2017.

### Cumulus grafting

To extend the time window for cumulus grafting experiments, embryos of the same cocoon were incubated at different temperatures (20 **°**C, 22 **°**C, 25 **°**C, 27 **°**C). This allowed me to obtain cumulus migration stages (embryonic stage 5, see below) over a time period of 5 days.

For cumulus grafting experiments, 50–100 stage 5 embryos were dechorionated using 2.8% hypochlorite solution (DanKlorix). The dechorionisation step was mandatory for the survival of the embryos. Dechorionated embryos were washed several times with distilled water. The embryos were attached to the membrane of a single cell culture dish (see above) and covered with Halocarbon oil 700 (SIGMA). With the help of SuperFine Vannas Scissors (World Precision Instruments), the vitelline membrane of the donor embryo was cut in a region close to the cumulus. A pulled glass capillary was used to cut out the cumulus of the “donor embryo”. By squeezing the vitelline membrane of the “donor embryo”, it was possible to transfer a small droplet of the perivitelline fluid together with the excised cumulus into the surrounding oil. Using the SuperFine Vannas Scissors, the vitelline membrane of a nearby embryo (the “cumulus acceptor”) was cut at a position that was opposite to the side of the already migrating endogenous cumulus. As the migrating cumulus bulges the overlaying ectoderm in a kind of triangular shape, the direction of the movement of the cumulus was traceable in most embryos. With the help of the glass capillary, the droplet of perivitelline fluid containing the excised cumulus was carefully guided through the oil towards the cut in the vitelline membrane of the “cumulus acceptor”. The droplet within the oil (containing the cumulus of the “donor embryo”) was fused with the perivitelline fluid of the “acceptor embryo” by simultaneously touching both fluids with the glass capillary. After droplet fusion, the “donor cumulus” was carefully inserted into the perivitelline space of the “acceptor embryo” and was moved a bit downward to clamp it between the vitelline membrane and the ectoderm of the “acceptor embryo”.

### Bead transplantation

Recombinant human BMP4 protein (Thermo Fisher Scientific; PHC9534) was reconstituted in 0,1% bovine serum albumin (BSA). Affi-Gel Blue beads, diameter 150–300 μm (Bio-Rad; #1537301), were washed three times in PBS. Prior to implantation, around 100 washed beads were incubated for 1.5 h (at room temperature) in 5 μl of 100 μg/ml recombinant human BMP4 protein or 0.1% BSA.

Prior to bead transplantation, embryos were prepared as described in the section “cumulus transplantation” and covered with Halocarbon oil 700 (SIGMA). The soaked BMP4 or BSA (as a control) beads were pipetted into the oil, and single beads were separated within the oil using a pulled glass capillary. Using the SuperFine Vannas Scissors, the vitelline membrane of an embryo was cut at a position that was opposite to the side of the already migrating endogenous cumulus. Single beads were guided through the oil towards the cut in the vitelline membrane. With the help of the pulled glass capillary, a small cut was made in the ectoderm of the germ-disc. The bead was inserted into this cut and was pushed below the ectoderm.

### Embryo fixation, in situ hybridisation, durcupan sections and pMad antibody staining

Embryo fixation was performed as described for *Parasteatoda tepidariorum* (Pechmann et al. 2017) with minor modification. Fixed embryonic material is available upon request.

For the fixation of cumulus – or bead – transplanted embryos, about 3 μl of 10% formaldehyde was injected to the perivitelline space. Excess oil was poured from the lummox dish after an incubation time of 1–2 h. Using a scalpel, the membrane with the attached embryos was cut out from the lumox dish. The embryos (still glued on the membrane) were placed upside-down on the aqueous phase of the fixative (5% formaldehyde in PBS) and were covered with 100% heptane. After 30 min, most of the embryos were washed of the membrane and were transferred to fresh fixative (3 ml PBS, 3 ml 10% formaldehyde and 6 ml heptane). Embryos were fixed on a shaker overnight at room temperature and 50 rpm.

In situ hybridisation was performed as described for *C. salei* (Prpic et al. 2008) with minor modification (no proteinase K treatment; doubling of buffer volumes; duration of washing steps was doubled; final overnight washing step in 15 ml PBST).

Durcupan sections (20 μm cross-sections) of stage 5 embryos and pMAD antibody staining were performed as described in Pechmann et al. 2017. For pMad antibody staining, cumulus or bead transplanted embryos were fixed 15–20 h after transplantation.

## Results and discussion

### *A. geniculata* as a model system in the lab

*A. geniculata* is a huge and colourful tarantula (body length of around 8 cm and a leg span of around 20 cm; see Fig. 1b and Fig. S1a,b) from Brazil. The spider should be handled with care as the behavioural character can vary from shy to very aggressive, and urticating hairs are regularly used as a defence mechanism.

Regarding the long generation time of Mygalomorphs, it is challenging to analyse evolutionary developmental questions in this group of spiders. However, *A. geniculata* females produce a huge number of eggs/cocoon (between 1000 and 2000), and the spiders can be maintained and raised in the lab throughout the year. In addition, transcriptomic data (this work and Sanggaard et al. 2014), as well as a draft assembly of the sequenced genome of *A. geniculata*, are available (Sanggaard et al. 2014), and in situ hybridisations were previously established in embryos of this spider species (Pechmann and Prpic 2009). Finally, the big size of the embryos makes them amenable to transplantation experiments (this work). These facts compensate for the long generation time of this spider species and allow the analysis of evolutionary developmental questions.

Depending on temperature and food supply, females reach adulthood after 3–5 years. In contrast to the males, which reach adulthood after 3–4 years and only live for an additional year, the females can reach an age of more than 10 years. During that time the females moult regularly (on average once per year) to regenerate lost hairs or appendages. As transferred sperm is lost during the moulting process, the females have to be mated again. However, a spider culture of around 30 individuals with 10–20 adult females and males of different age ensures that several cocoons can be obtained every year.

After their final moult, male spiders show a clear sexual dimorphism (Fig. S1a,b). The distal part of the pedipalp has developed into a copulatory organ, the bulb (Fig S1c,d). Prior to copulation, males produce a specialized web that is used to attach a drop of sperm. This drop of sperm is then actively taken up into the bulb. In addition to the pedipalpal bulb, the first walking leg has developed a tibial apophysis (Fig. S1e). During mating, the male (spider to the right in Fig. 1b) uses the tibial apophysis to lift up the female. This enables the male to reach the genital opening of the female on the ventral side of the opisthosoma and to insert the bulb of the pedipalp at the right angle to transfer the stored sperm. The female is able to store the sperm for several months in the receptaculum seminis (Fig. S1f).

A complicated mating dance precedes the copulation, and it can take minutes to hours for the final step of sperm to transfer. Males that are stimulated by touching the silk or an exuvium of a female spider will start to send out species-specific vibrations. Females that are willing to mate will answer violently by tapping the ground (like a drum roll). This behaviour can also be used to check whether a female has reached adulthood.

After mating, females were regularly fed to ensure egg production, and cocoons were observed 10.5 weeks after mating (time between mating and cocoon production of four spider females was monitored). The timing of cocoon production may vary depending on the season, feeding status of the female, temperature and humidity. The cocoon production itself takes several days and involves the establishment of a giant bowl-shaped web (see Fig. 1c). The final cocoon has a ball like appearance (Fig. 1d,e) and is protected by the female. The size of the cocoon and the number of eggs depend on the size of the female and food supply. One of the cocoons obtained for this study contained around 2000 eggs (Fig. 1d–f).

### Embryonic development of *A. geniculata*

Several publications deal with the description of the development of Araneomorphae spider species (Mittmann and Wolff 2012; Turetzek and Prpic 2016; Wolff and Hilbrant 2011). Unfortunately, the embryonic staging varies across the different publications (e.g., 21 embryonic stages for *Cupiennius salei* vs. 14 embryonic stages for *P. tepidariorum* or hours after egg lay in *Pholcus phalangioides*). This fact makes it difficult to compare embryonic stages of different spider species. Nevertheless, stage 1 (early cleavage stages before contraction), stage 5 (cumulus migration), stage 8 (segmented germ band before appendage outgrowth) and stage 9 (appendages start to develop) are embryonic stages that are common to all spider species studied so far. These stages should be used as a landmark for staging spider species and have been used in this study to compare and align the development of *A. geniculata* embryos to other spider species, like *C. salei* or *P. tepidariorum* (Fig. 1a).

### Stage 1–3: energid cleavages, contraction and blastoderm

**Fig. 2:**
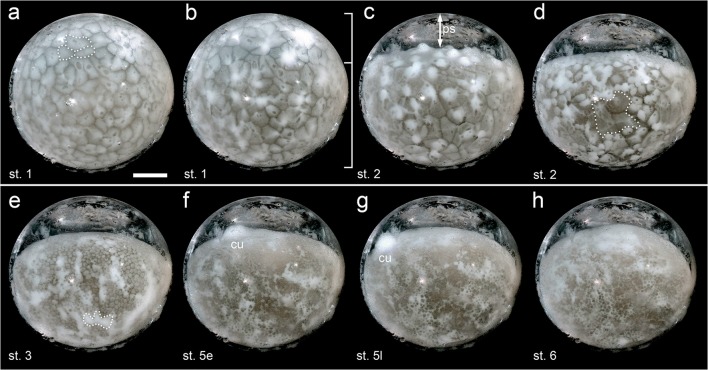
Embryonic development of *A. geniculata* (st. 1–6; side view). Stills from Movie S1. The chorion was not removed and can be recognized as whitish, granulated material on the surface of the vitelline membrane. **a** Embryo at mid stage 1. Some polygonal and ovoid shaped cortical fields are marked by the dotted line. **b** Cleavage energids reach the surface and fuse with the cortical fields. The lower two thirds of the egg seem to be more densely packed with yolk granules (indicated by the bracket, compare to Fig. S3a). **c** Embryo contraction is an indicator for the beginning of stage 2. The blastoderm contracts onto the densely packed yolk granules, which results in the appearance of the perivitelline space (ps). **d** Late stage 2. The blastoderm is still very irregular with cortical regions that are partially free of energids (dashed line in d). **e** Further asynchronous cell divisions and contraction of the large cortical fields (the development of the white area marked by the dashed line in **e** is shown in Fig. S4) are leading to a regular blastoderm that is interspersed with white spots. **f** and **g** Stage 5 is characterized by the appearance and the migration of the cumulus (cu). **h** The cumulus disappears at the beginning of stage 6 and the formation of the axially symmetric spider embryo is initiated. Scale bar is 500 μm

Freshly laid eggs are very fragile and surrounded by a fluid that is absorbed during the first hour of development (Foelix 2011). This absorbance process leads to the formation of the chorion that surrounds the egg and most likely protects the eggs against desiccation and bacterial infections (e.g., Makover et al. 2019). The chorion is irregularly distributed on the surface of the vitelline membrane and can be recognized as a whitish and granulated material (see Fig. 2 and Movies S1, S2). In contrast to many other spider species (e.g., *P. tepidariorum*), the chorion of *A. geniculata* eggs is quite transparent, and it is not necessary to remove the chorion or to immerge the eggs in oil to stage the embryos.

Eggs that were removed a few hours after egg deposition did not survive. Only around 24 h after egg deposition it was possible to carefully remove single eggs from the cocoon and to analyse the very early steps of embryogenesis (see below).

In contrast, most of the eggs of a cocoon that was opened 3 days after egg lay could easily be separated from each other. These embryos were still at stage 1 of development, and it was possible to observe the cortical periplasm that was divided into ovoid or polygonal fields (dotted line in Fig. 2a).

During stage 1, gravity is leading to an irregular distribution of larger and denser yolk granules within the egg. As a result, the upper third of the stage 1 pre-contraction egg is more transparent than the lower two thirds of the egg (see brackets in Fig. 2b and the dotted line in Fig. S3a, Movies S1 and S2). This phenomenon has been described for other spider species as well, and it has been suggested that this is common to large eggs (Holm 1940). However, the subdivision of a region of more densely packed yolk granules seems not to be connected to any polarity of the egg as the process can be reverted as soon as one rotates the egg upside-down (own observation; (Holm 1952)). In addition to *A. geniculata*, I also had access to very early embryonic stages of *Brachypelma albopilosum,* another theraphosid spider species. Time-lapse imaging of *B. albopilosum* embryos that were at the beginning of stage 1 (around 24 h after egg deposition) revealed that the establishment of these cortical polygonal fields occurred several hours after egg lay (see Movie S2 and Movie S3). The nature and the content of the cortical polygonal fields are not well described. However, these fields also develop in other spider species, and it has been suggested that these fields are required for the cellularization process that occurs at the end of stage 1 (Balbiani 1873; reviewed in Holm 1940).

Nuclei with surrounding cytoplasm (so-called cleavage energids) are the characteristics of the embryonic stage 1. The energids start to divide in the centre of the egg and reach the periphery of the egg at the end of stage 1 (Fig. 2b; Fig. S2a; Movies S1, S2 and S4). In spiders, cytoplasmic strands connect the cleavage energids to the cortical fields (reviewed in Schwager et al. 2015), and these strands seem to be required to bring the energids from the centre towards the surface of the egg. If this is an active (via contraction of the strands) or passive (flexible strands under tension) process is unknown.

At the end of stage 1, the energids have reached the surface of the egg, and each energid fuses with several polygonal cortex fields (see Fig. 2b; Fig. S2a; Movies S1, S2 and S4). As soon as the energids have invaded the cortex, polygonal cortex fields and energids divide synchronously (see Movie S1), and it is likely that the cortex is providing the vast majority of cell membrane material that is required during the cellularization process in *A. geniculata*. In true spiders like *P. tepidariorum*, cellularization is completed before the energids reach the surface of the egg (e.g., Kanayama et al. 2010). However, it remains to be elucidated if this also holds true for tarantula eggs.

The beginning of embryonic stage 2 is characterized by the contraction of the embryo (Fig. 2c; Fig. S2b; Movies S1, S2). This contraction leads to the formation of a broad space in the upper third of the egg that is filled with perivitelline fluid.

By comparing eggs of *B. albopilosum* with eggs of *A. geniculata*, I could observe two modes of the contraction process. In *A. geniculata* the cleavage energids reached the cortex all around the egg, and the established blastoderm contracted on top of the densely packed yolk granules (see Fig. 2c, Movie S1). This was in contrast to *B. albopilosum* embryos in which the cleavage energids reached the surface of the egg only in the lower two third of the egg (in the region of the densely packed yolk granules; see Fig. S3a,b and Movie S2). Thereafter, the upper, energid free cortex, collapsed on top of energids that arrested at the surface of the densely packed yolk mass (see Fig. S3b,c and Movie S2).

As the polygonal cortical fields are very fragile structures, single or groups of fields were regularly damaged while handling stage 1 embryos. However, this damage of the cortex allowed me to see below the cortex, and I was able to image the movement of the energids along the connecting strands during the contraction phase in *B. albopilosum* embryos (see Fig. S3e,e’ and Movies S4 and S5).

**Fig. 3:**
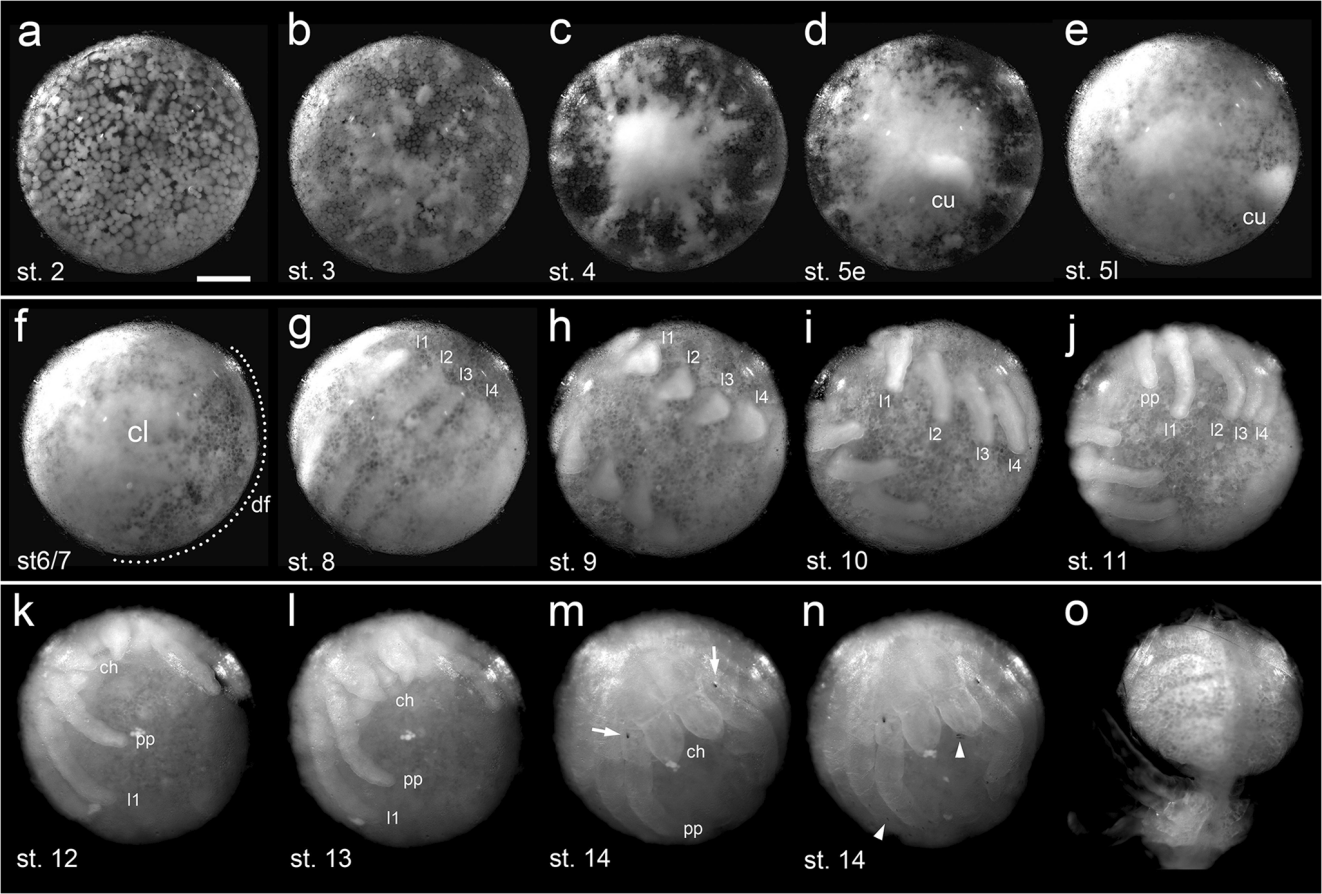
Embryonic development of *A. geniculata* (st. 2 until hatching; top view). Stills from Movie S7 (**a–j**) and Movie S10 (**k–o**). **a** Embryo at late stage 2 showing irregularly distributed blastoderm cells. **b** A regular blastoderm has formed that is interspersed with white spots (compare to Fig. 2e). **c** At stage 4 many cells aggregate and form the “primary thickening” that seems to be the source for the developing cumulus and for most of the cells that gastrulate during stage 5. **d** and **e** The cumulus (cu) appears at early stage 5 and migrates from the region of the primary thickening towards the rim of the “germ-disc” (please note that in tarantula embryos a distinct germ-disc is not visible via bright field imaging). **f** The cumulus disappears at the beginning of stage 6. At the same time the dorsal field (df) gets established and extensive morphogenetic rearrangements are leading to the formation of the axially symmetric germ band. The region of the primary thickening has developed into the caudal lobe (cl). **g** A clearly segmented germ-band is present at stage 8. **h–l** Embryonic development proceeds. Appendages start to grow out and elongate to their final length, posterior segments are added and dorsal closure is completed. See text and Fig. 5 and Fig. 6 for more details. **m,n** Ventral closure and the pigmentation of the egg tooth (arrow in **m**), bristles and of the “false pincer” of the chelicera (arrowheads in **n**) occurs at embryonic stage 14. **o** The postembryo hatches from the egg. Abbreviations: st. 5e, stage 5 early; st. 5l, stage 5 late; cu, cumulus; df, dorsal field; l1-l4, walking legs; pp, pedipalp; ch, chelicera. Scale bar is 500 μm

At stage 2, blastodermal cells are large and irregularly distributed (Fig. 2c,d; Fig. 3a; Movie S1, S2, S4, S7). In addition, not every polygonal field of the cortex has been occupied by an energid (dashed line in Fig. 2d and boxed region in Fig. S4a–d). Asynchronous cell divisions lead to stage 3 embryos that show a relatively regular distribution of small blastodermal cells (Fig. 2e; Fig. 3b; Movie S1 and S7). At the same time, contraction of cortical fields leads to the formation of big white patches that can be observed throughout the blastoderm (the development of the white patch marked by dashed line in Fig. 2e is shown in Fig. S4).

At stage 3, some cells appear whiter than others, and this difference in colour is sometimes connected to a different cell fate. Movie S6 shows a group of cells of white appearance that start to become migratory during stage 5 of development.

### Stage 4–5: primary thickening, germ-disc and cumulus migration

During stage 4, hundreds of cells accumulate and form a white disc of around 500 μm in diameter (Fig. 3c; Movie S7 and S8). A similar cell accumulation is visible in a variety of spider species and was named primitive plate or primary thickening in former publications (e.g., Holm 1952; Wolff and Hilbrant 2011). In the following, I refer to this accumulation of cells as the primary thickening.

The primary thickening is the characteristic structure of a stage 4 embryo, and it seems to be the main source of cells that gastrulate and spread over the embryo during embryonic stage 5. In addition the migratory cumulus arises in the centre of the primary thickening (Fig. 3d; Movies S7 and S8).

**Fig. 4:**
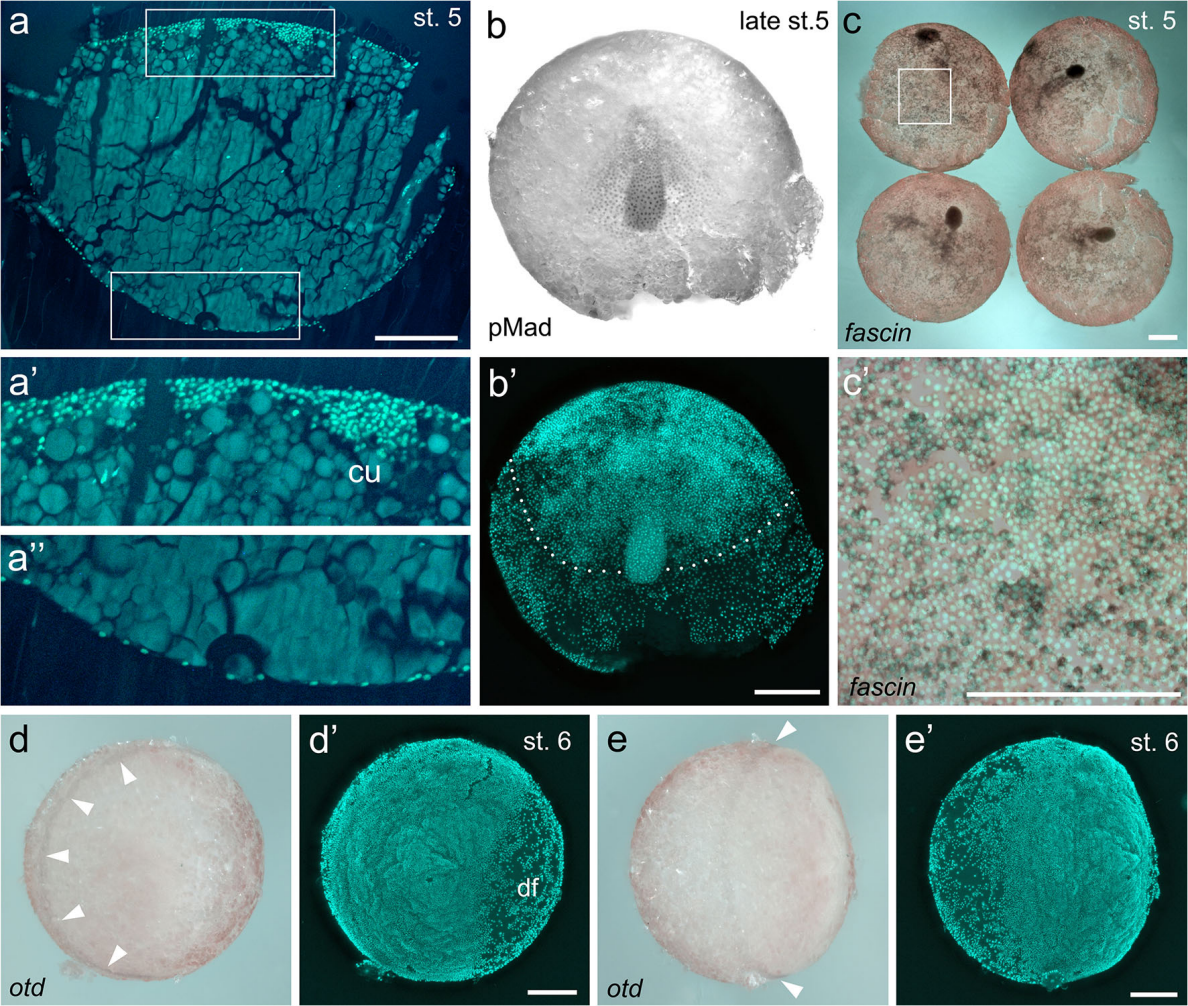
The cumulus and the germ-disc of *A. geniculata* embryos. **a–a”** Cross-section through a Sytox green-stained stage 5 embryo. Boxed regions in **a** are magnified in **a’** and **a”**. The cumulus (cu) consists out of hundreds of cells. While the upper, embryonic half of the embryo is multi-layered (**a’**), the lower, ab-embryonic half of the embryo is single layered (**a”**). **b–b’** Visualization of the active BMP signalling pathway (via pMad antibody staining). Phosphorylated Mad protein is only detectable in ectodermal cells surrounding the migrating cumulus. The border between the embryonic and ab-embryonic half of the embryo, the equator, is indicated by the dashed line in **b’**. **c–c’***A. geniculata fascin* is expressed in the cumulus (**c**). In addition *fascin* is expressed in cells that are likely to be gastrulating endodermal cells (boxed region in **c** is magnified in **c’**). **d,e** The germ-disc has opened up at stage 6 of embryonic development and the dorsal field (df) has been established. The anterior marker *otd* is expressed in a faint anterior “open-circle” (see arrowheads in **d** and **e**). Scale bar is 500 μm

While a distinct radially symmetric germ-disc is visible at stages 3–5 in spider species like *P. tepidariorum* or *Zygiella x-notata* (Chaw et al. 2007; Mittmann and Wolff 2012), no distinct germ-disc is visible in tarantula embryos. This is similar to *C. salei* in which a germ-disc is first visible at stage 5 when gastrulating cells only invade the embryonic half of the egg leading to a distinct border (termed the equator) between the embryonic and ab-embryonic half of the egg (Wolff and Hilbrant 2011). Also in *A. geniculata*, gastrulating cells seems to primarily invade the embryonic half of the egg (the germ-disc), and an equator-like rim of the disc is visible at stage 5 of development (dotted line in Fig. 4b’). Physical sectioning of stage 5 embryos revealed that while the upper, embryonic germ-disc is multi-layered, the lower ab-embryonic half of the egg is single-layered (Fig. 4a–a”). Further evidence for the presence of a germ-disc, albeit not visible with bright field imaging, is the fact that the cumulus migrates only until it has reached the equator (Fig. 2f,g; Fig. 3d,e; Movies S1 and S7-S9).

In spiders, the cumulus is a group of mesenchymal cells that function as a migratory cell cluster to break the radial symmetry of the germ-disc. The cumulus is likely an evolutionary novelty of chelicerates, and it has been observed in a variety of chelicerate species (reviewed in Schwager et al. 2015 and Hilbrant et al. 2012). In spiders, it has been shown that the cumulus is required to induce (via the activation of the BMP signalling pathway, see below) the so-called dorsal field (Akiyama-Oda and Oda 2006; Oda and Akiyama-Oda 2008). In *A. geniculata*, the cumulus is about 250 μm in diameter and consists of hundreds of cells (Fig. 2f,g; Fig. 3d,e; Fig. 4a–c). As in *P. tepidariorum*, the cumulus expresses the cumulus marker *fascin* (Akiyama-Oda and Oda 2010), and it activates the BMP signalling pathway (Akiyama-Oda and Oda 2006) in overlaying ectodermal cells (Fig. 4b,c). *A. geniculatafascin* expression is also visible in single cells and in clusters of cells (Fig. 4c’) throughout the germ-disc. It is likely that these cells are migrating gastrulating endodermal cells as it was shown for *fascin* positive cells in *P. tepidariorum* (Akiyama-Oda and Oda 2016).

### Stage 6–8: dorsal field and germ-band formation

In *P. tepidariorum* the gene *orthodenticle* (*otd*) is a good marker for anterior tissue identity, and it is expressed all around the anterior rim of the germ-disc (e.g., Akiyama-Oda and Oda 2003; Pechmann et al. 2009). When the cumulus reaches the rim of the germ-disc, it initiates the opening of the disc via the establishment of the dorsal field. This is also reflected by the expression of *otd*, which forms an “open” circle (Akiyama-Oda and Oda 2003; Pechmann et al. 2009). Also, in *A. geniculata otd* is expressed as a faint “open” circle at stage 6 of embryonic development (Fig. 4d,e), and the cumulus seems to be required to induce the dorsal field (Fig. 3f; Fig. 4d’; Movie S7). At the beginning of stage 6, the cells of the cumulus lose contact to each other, and they spread over the dorsal field of the embryo (best visible in Movies S1 and S9). So far, the final fate of the cumulus cells is still unclear. Labelling experiments and molecular data suggest that cumulus cells as well as cells within the dorsal field possess an endodermal cell fate (Akiyama-Oda and Oda 2003; Edgar et al. 2015; Feitosa et al. 2017; Holm 1952), and the spreading of the cumulus cells over this area in *A. geniculata* supports this idea. However, the recent finding that *twist* (a marker for mesodermal cells) is also expressed within the developing cumulus in *P. tepidariorum* suggests a mesendodermal nature of the cumulus and indicates that the situation might be more complicated (Pechmann et al. 2017).

**Fig. 5:**
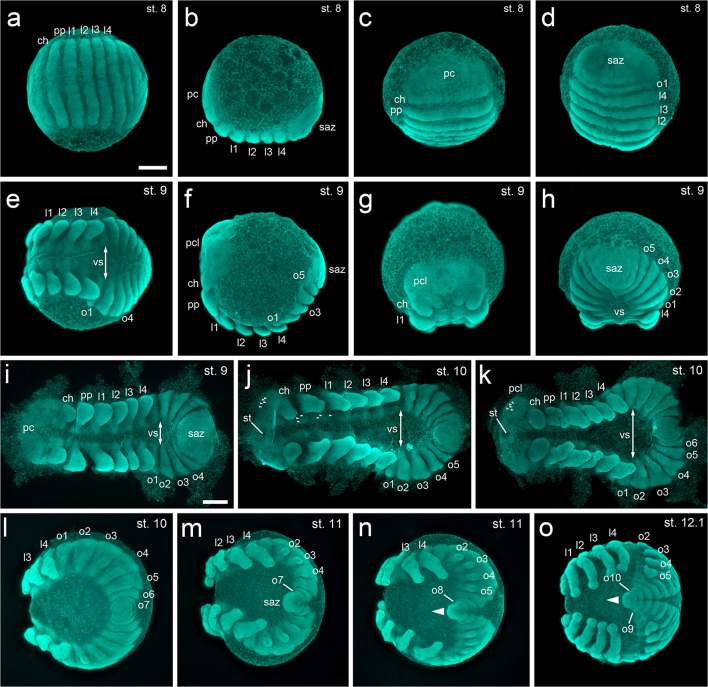
Embryonic development of *A. geniculata* (stages 8–12). **a–d** All prosomal segments are established and patterned at stage 8 (compare to Fig. 7a). The pre-cheliceral lobe and the segment addition zone have a similar size and shape. **e–i** At stage 9, appendages start to grow out. The left and the right half of the embryo are clearly separated from each other, and the ventral sulcus is widest in the anterior region of the opisthosoma (indicated by the double headed arrow in **i–k**). **j,k** The development of the stomodeum and the nervous system (arrowheads in j and k) is initiated. The distal parts of the pedipalps and walking legs are more pointed. The ventral sulcus has reached its maximum width in the region of the first and second opisthosomal segment. As a result, the posterior of the embryo has a “horseshoe” appearance. **l,m** Special focus on the transformation of the segment addition zone. From stage 10 to stage 12 the posterior of the embryo transforms into a small tube that starts to grow towards the anterior (arrowhead in **n** and **o**). At the same time appendage buds appear on o2-o5 (see Fig. 6 for more details). All embryos were fixed and stained with the fluorescent nuclear dye Sytox green. Ventral view (**a,e**); lateral view (**b,f**); anterior view (**c,g**); posterior view (**d,h**); ventral-posterior view (**l–o**); flat mounted embryos (**i–k**). Abbreviations: ch, chelicera; l1-l4, walking leg segments 1–4; o1–10, opisthosomal segments 1–10; pc, pre-cheliceral region; pcl, pre-cheliceral lobe; pp, pedipalp; saz, segment addition zone; st, stomodaeum; vs, ventral sulcus. Scale bar is 500 μm

It has been shown that convergent extension mechanisms are the main driver to form an axially symmetric spider embryo out of a radially symmetric germ-disc (Kanayama et al. 2011). In *A. geniculata*, the differences between a stage 6 and a stage 7 embryo are only subtle (stage 7 is more triangular shaped, see Movie S7 and the respective figure legend), and it is a smooth transition to form the segmented stage 8 embryo (Fig. 3f,g). Stage 8 is completed when prosomal segments are clearly segmented and prosomal appendage buds are about to form (Fig. 3g; Fig. 5a–d). The expression of the segment polarity gene *engrailed* (*en*) reveals that prosomal segments are well established at stage 8 of embryonic development (Fig. 7a).

### Stages 9–11: appendage formation, inversion and a switch in the segment addition zone

Embryonic stage 9 is characterized by the outgrowth of the prosomal appendages (Fig. 3h; Fig. 5e–i). At the beginning of stage 9, broad blocks of segments showing continuous *en* expression are sequentially added from the segment addition zone (saz) (Fig. 7e). At the end of stage 9, the first sign of the ventral sulcus (ventral opening of the embryo which initiates the process of inversion (described in e.g., Crome 1963; Wolff and Hilbrant 2011)) is visible (Fig. 5e,h,i), and *en* stripes of the opisthosomal segments 1–3 are split into a left and a right stripe per segment (Fig. 7b). Only at the anterior end of the saz, continuous, semi-circular expression of *en* was still detectable (o4 segment in Fig. 7b). In spiders that belong to the Entelegynae (like *C. salei* or *P. tepidariorum*), inversion (the ventral splitting of the embryo into a left and a right body half) is a process that is relatively uniform along the anterior-posterior body axis. In these spiders, the ventral sulcus is widest in the median region of the germ-band, and it converges pointedly towards the posterior of the embryo (e.g., Mittmann and Wolff 2012; Wolff and Hilbrant 2011). This is in contrast to the inversion process of *A. geniculata* in which the ventral sulcus broadens during embryonic stages 9 and 10 and is widest in the second opisthosomal segment and converges pointedly towards the anterior of the embryo (Fig. 5i–k; Fig. 7c,f,g; Fig. 10a). At the end of stage 10 the opisthosoma has a horseshoe-like appearance (Fig. 5k,l). This horseshoe-like split of left and right side of the opisthosoma has been noted in other mygalomorph and chelicerate species before (reviewed in Setton et al. 2019).

Connected to the described shape changes within the opisthosoma, there seems to be a switch in the segmentation process as well. While complete and continuous segments are added one after the other from the posteriorly located saz at embryonic stages 8 and 9 (Fig. 7e), segments seem to be added in a ring and wave like fashion to the left and right body half during embryonic stage 10 (Fig. 5k,l; Fig. 7c,g; Fig. 10a; Fig. S7c). The ring- and wave-like addition of segments of stage 10 *A. geniculata* embryos is also evident in the expression of segmentation genes. It was shown that in the saz of *P. tepidariorum*, *caudal* (*cad*) might be required for the activation and maintenance of the expression of the pair-rule gene *even-skipped* (*eve*) (Schönauer et al. 2016). Also in *A. geniculata cad* and *eve* seem to be expressed in overlapping domains within the saz (Fig. 7i and k). Furthermore, similar to *P. tepidariorum, A. geniculata eve* also shows a wave like cyclic expression (compare Fig. 7j,k) in the saz. In addition, the pair rule-gene *hairy* (*h*) is also expressed in ring-like domains within the newly formed segments (Fig. 7l).

Wave- and ring-like addition of segments from the saz has also been observed in arthropods like myriapods (e.g., Chipman and Akam 2008; Janssen et al. 2011). This suggests that this kind of genetic patterning during segment addition may represent an ancestral feature of arthropod segmentation.

**Fig. 6:**
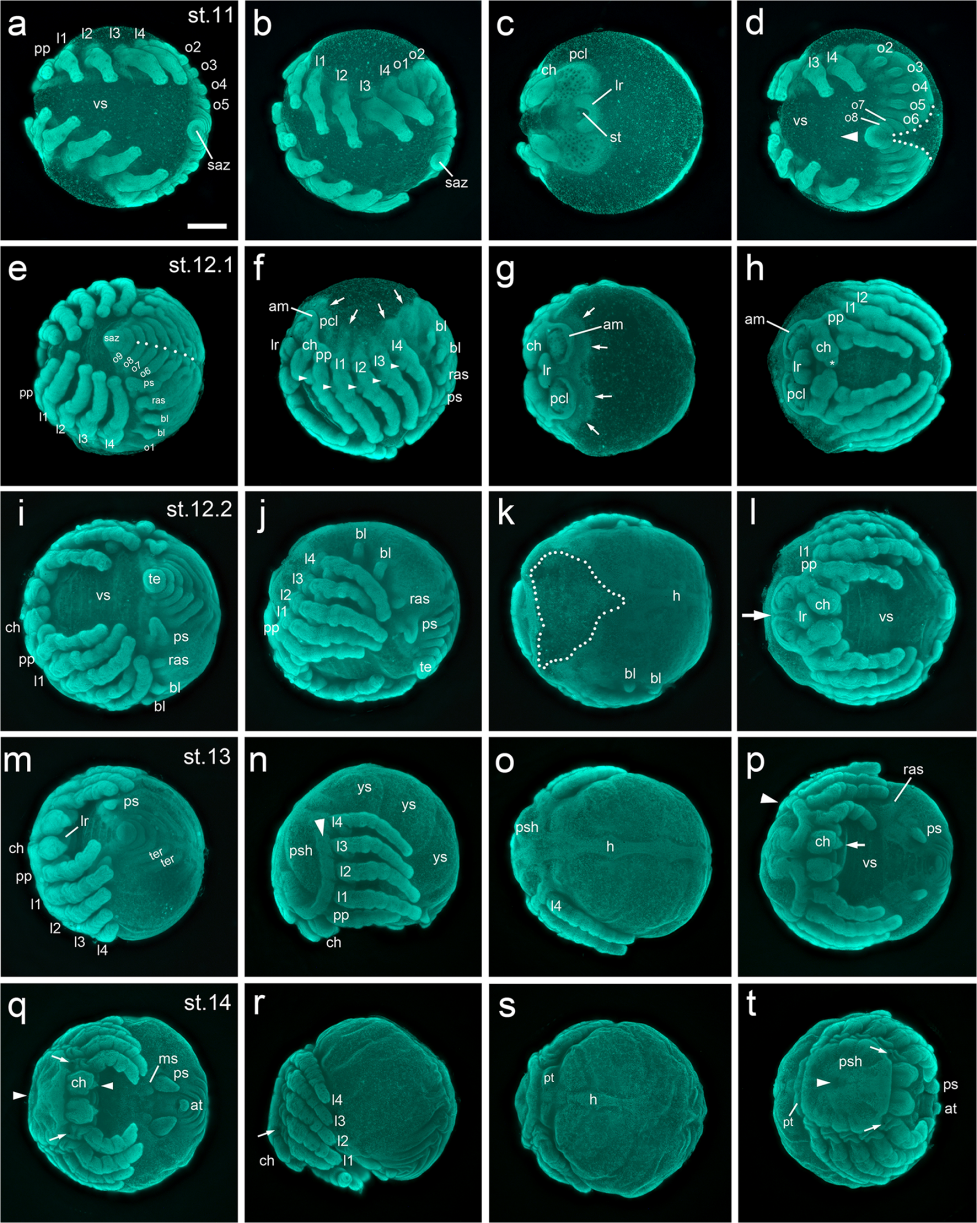
Embryonic development of *A. geniculata* (stages 11–14) **a–d** At stage 11 the saz is pointing towards the anterior (arrowhead in **d**). The ventral sulcus has a triangular shape with its narrowest point in the cheliceral segment. The labrum has started to form. Appendage buds on opisthosomal segments 2–5 are clearly visible. **e–h** Dorsal closure starts at stage 12.1 (see arrows in **f** and **g**). The left and right side of the opisthosoma fuse along the dorsal midline (compare dotted line in **d** and **e**). Endites are visible at the base of the appendages (arrowheads in **f**). The brain is framed by the anterior margin. The chelicera is clearly divided into the base and the fang (see asterisk in **h**). **i–l** At stage 12.2 dorsal closure is at an advanced stage (dotted line in **k** indicates the dorsal region that has not closed yet). The two head lobes start to fuse along the midline (arrow in **l**), and the chelicere of the left and right body half start to close over the labrum (**l**). During stage 12, only the appendage buds on the second and third (the book lungs) and the fifth (the posterior spinneret) opisthosomal segment grow and develop further. The rudimentary bud on o4 degenerates until the end of embryonic development. **m–p** Ventral closure starts at stage 13. The labrum sits below the cheliceres. Dorsal closure has led to the formation of the prosomal shield and the heart. The rim of the prosomal shield is pronounced (arrow head in **n**). The opisthosoma is divided into big “yolk sacks”. The fang of the chelicera is very pointed (arrowhead in **p**). **q–t** Stage 14. Ventral closure has led to the formation of a flat prosoma. The egg tooth (arrows in **q**, **r** and **t**) is visible and the petiolus has formed. The posterior spinnerets are in a ventral posterior position, close to the anus. Median spinnerets have formed. The apodeme is visible in the centre of the prosomal shield (arrowhead in **q** and **t**). All embryos were fixed and stained with the fluorescent nuclear dye Sytox green. Abbreviations: at, anal tubercle; am, anterior margin; bl, book lung; ch, chelicera; h, heart; l1-l4, walking legs; lr, labrum; ms, median spinneret; o1–9, opisthosomal segments 1–9; pcl, pre-cheliceral lobe; pp, pedipalp; ps, posterior spinneret; psh, prosomal shield; pt, petiolus; ras, rudimentary anterior spinneret; saz, segment addition zone; st, stomodaeum; te, telson; ter, tergite; vs, ventral sulcus; ys, yolk sack. Scale bar is 500 μm

Between stage 10 and stage 11 the posterior end of the spider embryo passes through another shape change. Similar to what has been described for the posterior end of Mesothelae (Yoshikura 1954), other mygalomorph spiders like *Atypus karschi* (Crome 1963; Yoshikura 1958) as well as for representatives of haplogyne spiders like *P. phalangioides* or *Segestria bavarica* (e.g., Crome 1963; Holm 1940; Turetzek and Prpic 2016; Wolff and Hilbrant 2011), the segment addition zone starts bulging outwards, and a tube-shaped structure grows into an anterior direction (Fig. 5m–o; Fig. 6d; Fig. 10a; Fig. S7d). This tube-like structure has been named the “post-abdomen” (Holm 1940) and the most posterior segments are added sequentially from this tube. This “downturned” opisthosoma is a plesiomorphic character of Arachnopulmonata (reviewed in Setton et al. 2019; Turetzek and Prpic 2016).

In *A. geniculata* and *B. albopilosum* embryos, the “post-abdomen” is never as prominent as in *A. karschi*, *P. phalangioides or S. bavarica*. However, as the “post-abdomen” is present in all big branches of the Araneae, patterning the posterior segments within a tube-like structure, is likely to resemble the ancestral state of posterior segment addition within spiders (reviewed in Turetzek and Prpic 2016; Wolff and Hilbrant 2011).

**Fig. 7:**
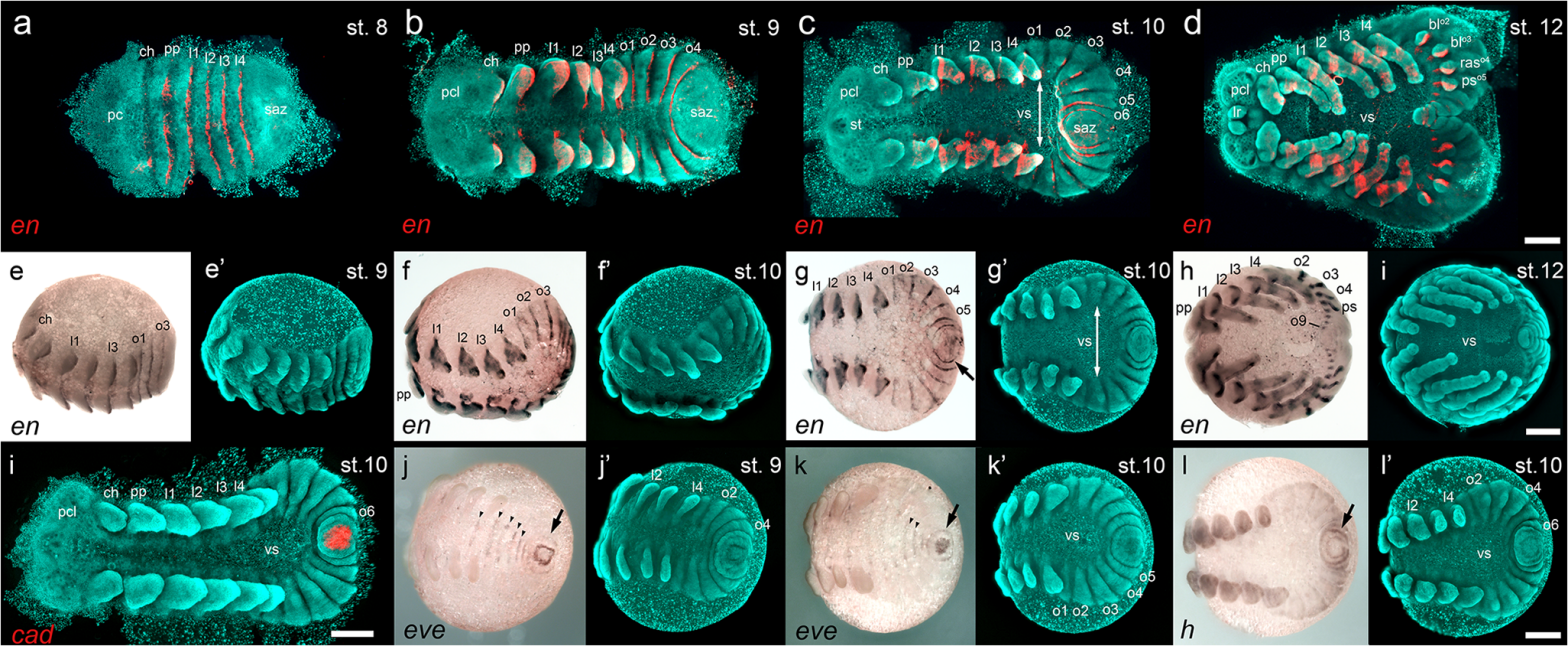
Marker gene analysis in embryos of *A. geniculata*. **a–h** Segmental expression of the segment polarity gene *engrailed* at stages 8–12. The double headed arrow indicates the width of the ventral sulcus in **c** and **g’**. **i** Posterior expression of *cad*. **j–l** Wave like posterior expression of the pair-rule genes *eve* and *h* are indicated by the arrows in **j**, **k** and **l**. Segmental expression of *eve* in the posterior region of the ventral sulcus is indicated by the arrow-heads in **j** and **k**. **a–d**, **i** and **m** are flat mounted embryos with false colour overlays of the indicated gene expression pattern. Abbreviations: bl^o2^, book lung on opisthosomal segment 2; bl^o3^, book lung on opisthosomal segment 3; ch, chelicera; l1-l4, walking legs; lr, labrum; o1–9, opisthosomal segments 1–9; pc, pre-cheliceral region; pcl, pre-cheliceral lobe; pp, pedipalp; ps^o5^, posterior spinneret on opisthosomal segment 5; ras^o4^, rudimentary anterior spinneret on opisthosomal segment 4; saz, segment addition zone; st, stomodaeum; vs, ventral sulcus. Scale bar is 500 μm

Going from stage 8 to stage 10, the anterior of the tarantula embryo also changes its morphology. The precheliceral region is very uniform during stages 8 and 9 (Fig. 5c–i; Fig. 7a,b). As soon as the stomodeum (at stage 10) and the labrum (at stage 11) are established, the pre-cheliceral region gets divided into a left and right pre-cheliceral lobe (Fig. 5j,k; Fig. 6c). At stage 10 and 11, small indentations within the pre-cheliceral lobes and along the ventral neuro-ectodermal tissue (Fig. 5j,k) indicate that neurogenesis has started. In addition to the establishment of the bi-lobed labrum also the appendage buds on the opisthosomal segments 2–5 (Fig. 5m–o; Fig. 6a,d; Fig. S7d) have developed at stage 11. These appendage buds will develop into the respiratory system (the book lungs) and the spinnerets. While the book lungs will develop from the buds on opisthosomal segments 2 and 3, the spinnerets will develop from the bud on opisthosomal segments 5. In other spider species, the appendage bud on the fourth opisthosomal segments also develops into a pair of spinnerets (the anterior spinnerets). However, in Mygalomorphae the anterior spinnerets are missing and the appendage bud on opisthosomal segment 4 degenerates over time (reviewed in Foelix 2011; Pechmann and Prpic 2009; Pechmann et al. 2010). This degeneration process might be correlated to the missing expression of the leg gap gene *Distal-less* (*Dll*) (Pechmann and Prpic 2009; Pechmann et al. 2010). Indeed, in one cocoon of *B. albopilosum*, I observed some hatchlings that showed an additional spinneret anterior to the regularly established posterior spinnerets (Fig. S8d,e). Interestingly, also some fixed embryos of the same cocoon showed the spontaneous reactivation of the expression of *Distal-less* in one of the appendage buds of the fourth opisthosomal segment (Fig. S8f). It is unclear how this reactivation occurred and if this was due to a homeotic transformation or due to another developmental defect during earlier developmental stages.

### Stage 12: dorsal closure

The main characteristic of stage 12 is the process of dorsal closure. However, as many morphogenetic events take place during stage 12, I have divided this stage into two sub-stages (stages 12.1 and 12.2).

General features of stage 12 embryos are the fully elongated and segmented prosomal appendages (Fig. 6e–l). Furthermore, the chelicera is now divided into a proximal base and a distal fang (Fig. 6h), and endites are growing from the coxae of the pedipalps and the walking legs (arrow heads in Fig. 6f). These endites have been described before, and the endites on the walking legs might represent a kind of ground pattern of the arachnid post-cheliceral appendage (Pechmann and Prpic 2009). In comparison to the endites of the walking legs, the endites on the pedipalps are bigger and will develop into the gnathendite that is required for food processing. At the end of embryonic development, no endite-like structures will be visible at the proximal base of the walking legs. Similar to the situation observed in the spinnerets, only the endite of the pedipalp expresses *Distal-less* and does not degenerate during embryonic development (Pechmann and Prpic 2009). Finally, brain development has also progressed further, and the anterior margin is a very prominent structure (Fig. 6f–h) during stage 12.

At stage 12.1, dorsal closure has started mainly in the posterior, and the left and right body halves fuse along the dorsal midline (compare dotted line in Fig. 6d and e). In the anterior and all along the dorsal rim of the germ-band, some tissue is detectable that is growing dorsally to close the back (arrows in Fig. 6f,g).

At stage 12.2, dorsal closure is at an advanced stage, and mainly the dorsal region of the prosoma has to close (dotted line in Fig. 6k). In the opisthosoma, the heart tube is already visible. The two precheliceral lobes come together and fuse along the midline (arrow in Fig. 6l). The left and the right chelicera also come together more closely and start to close over the labrum. At the end of stage 12, the labrum is no longer bi-lobed. In a side view (Fig. 6j), it is obvious that the opisthosomal segments 2–5 have become enlarged. As more posteriorly located segments stay small and seem to get further reduced on the ventral side (see *engrailed* staining in opisthosomal segments 6–9 in Fig. 7h), the bud of the posterior spinneret has shifted towards a more posterior position.

### Stage 13: ventral closure

During stage 13, the ventral sulcus closes. Dorsal closure led to the formation of the prosomal shield and the heart tube (Fig. 6n,o). At the rim of the prosoma, a prominent ridge is visible (arrowhead in Fig. 6n and p). The fang of the chelicera is now very pointed (arrow in Fig. 6p). While the posterior spinnerets have grown to their final size, the anterior rudimentary bud of the spinneret is greatly reduced (ras in Fig. 6p). As opisthosomal segments 2–5 are greatly enlarged, the segments can be recognized as big “yolk sacs” (ys in Fig. 6n).

### Stage 14: petiolus, cuticle secretion, pigmentation and muscle twitching

Ventral closure brings the left and right spinneret buds into a ventral position. The spinnerets are now directly anterior to the anus (Fig. 6q). Median spinnerets are visible (Fig. 6q). The petiolus has formed out of the first opisthosomal segment (pt in Fig. 6s). As the yolk is now mainly located in the opisthosoma, a flat prosoma has developed. In the centre of the prosomal shield, an indentation, the apodeme (a muscle attachment point), has formed (arrowhead in Fig. 6q,t). Cuticle secretion has started and the egg tooth gets secreted from the base of the pedipalp (Movie S7 and S10; arrows in Fig. 6q,r,t; Fig. S6a,b).

During stage 14, a dorsally and a ventrally located tooth (the so-called false pincer (Galiano 1996)) are also secreted from the distal tip of the fang of the chelicera (Fig. S5). The bristles of the appendages and the false pincer of the chelicera become pigmented at the same time as muscle twitching starts (Movie S7 and S10).

### Postembryo

About 25 days after egg lay (analysed cocoons: *n* = 3; cocoons/embryos were incubated at 25 **°**C), embryonic development is completed, and the postembryo hatches from the egg (Movie S10; Movie S11; Fig. S6d–i). Hobbyists know this stage as “eggs with legs”. The hatching process itself is a relatively quick process (see Movie S11), and it takes only 30 min for the postembryo to completely hatch from the egg. In many spiders, hatching from the egg is connected to the first moulting from the embryonic cuticle (e.g., Mittmann and Wolff 2012; Wolff and Hilbrant 2011). I could not observe any embryonic cuticle in *A. geniculata*. Only the egg tooth (Fig. S6a–c) detached from the cuticle during the hatching process and was found at random positions within the stripped off eggshell or on the cuticle of the hatchling. At the beginning, the postembryo is shiny and mostly unpigmented (Fig. S6d–f) but is able to move its legs (see Movie S11). Some postembryos already feed on non-hatched eggs (not shown). A few days later, the cuticle of the 1st instar larva develops under the postembryonic cuticle. As the postembryonic cuticle is transparent, one can easily see the pigmented hairs and eyes through the cuticle (Fig. S6g–i). In addition, the fang of the chelicera has developed and projects into the dorsal tooth of the false pincer of the postembryonic cuticle (Fig. S6i). The postembryonic stage takes about 15 days (analysed cocoons: *n* = 3; cocoons/postembryos were incubated at 25 **°**C).

### 1st and 2nd instar larva

After moulting from the postembryonic cuticle, the first instar larva is the first stage that is able to actively walk around. This stage takes about 5 weeks (analysed cocoons: *n* = 2; cocoons/1st instar larva were incubated at 25 **°**C). During this stage the larva are still consuming the rest of the yolk that is present within the opisthosoma. The spinnerets are now completely segmented. Internal organs like the silk glands are developing, and a few days after moulting into the first instar larvae, the spinnerets are able to produce first silk strands. At the end of this larval stage, the cuticle of the 2nd instar larva is secreted underneath the cuticle of the 1st instar larva. Urticating hairs are developing, and as soon as these hairs are getting pigmented, a circular spot is visible on the opisthosoma (Fig. S6k).

Moulting from the first instar larval, cuticle is leading to a small spiderling (the second instar larva; Fig. S6l). This little spider is now able to feed on small insects like *Drosophila* and will cannibalize on its siblings.

### Axis duplication via cumulus grafts

Within the arthropods, axis patterning via a migrating signalling centre (the cumulus) seems to be an evolutionary novelty of chelicerates, and the presence of a cumulus in myriapods is still debated (reviewed in Hilbrant et al. 2012). As shown in this and several other studies, the cumulus of spider embryos migrates from the centre of the germ-disc towards the periphery of the disc and activates the BMP signalling pathway in ectodermal cells that are close to the cumulus (Fig. 4b, e.g. Akiyama-Oda and Oda 2006, 2003; Hilbrant et al. 2012; Oda and Akiyama-Oda 2008; Pechmann et al. 2017). This activation of the BMP signalling pathway in a subset of germ-disc cells is essential to break the radial symmetry of the disc and to initiate the formation of the dorsal field and the bilateral symmetry of the spider embryo (reviewed in Hilbrant et al. 2012; McGregor et al. 2008; Oda et al. 2019; Schwager et al. 2015). The importance of the cumulus and its signalling activity has been demonstrated in a variety of studies. First, the removal of the cumulus via extirpation, laser ablation or through genetic manipulation resulted in spider embryos that failed to break the radial symmetry of the germ-disc and failed to establish a dorsoventral body axis (Holm 1952; Oda et al. 2019; Pechmann et al. 2017). Second, inhibiting the activation of the BMP signalling pathway also inhibited the formation of an axially symmetric spider embryo (Akiyama-Oda and Oda 2006). Finally, the grafting of the cumulus led to the induction of a secondary body axis by inducing an ectopic dorsal field (Holm 1952; Oda et al. 2019). However, cumulus grafting experiments were so far only performed in araneomorph spider species (the funnel-web spider *Agelena labyrinthica* (Holm 1952) and the jumping spider *Hasarius adansoni* (Oda et al. 2019)), and functional analysis of the cumulus in basally branching spiders is entirely missing.

**Fig. 8:**
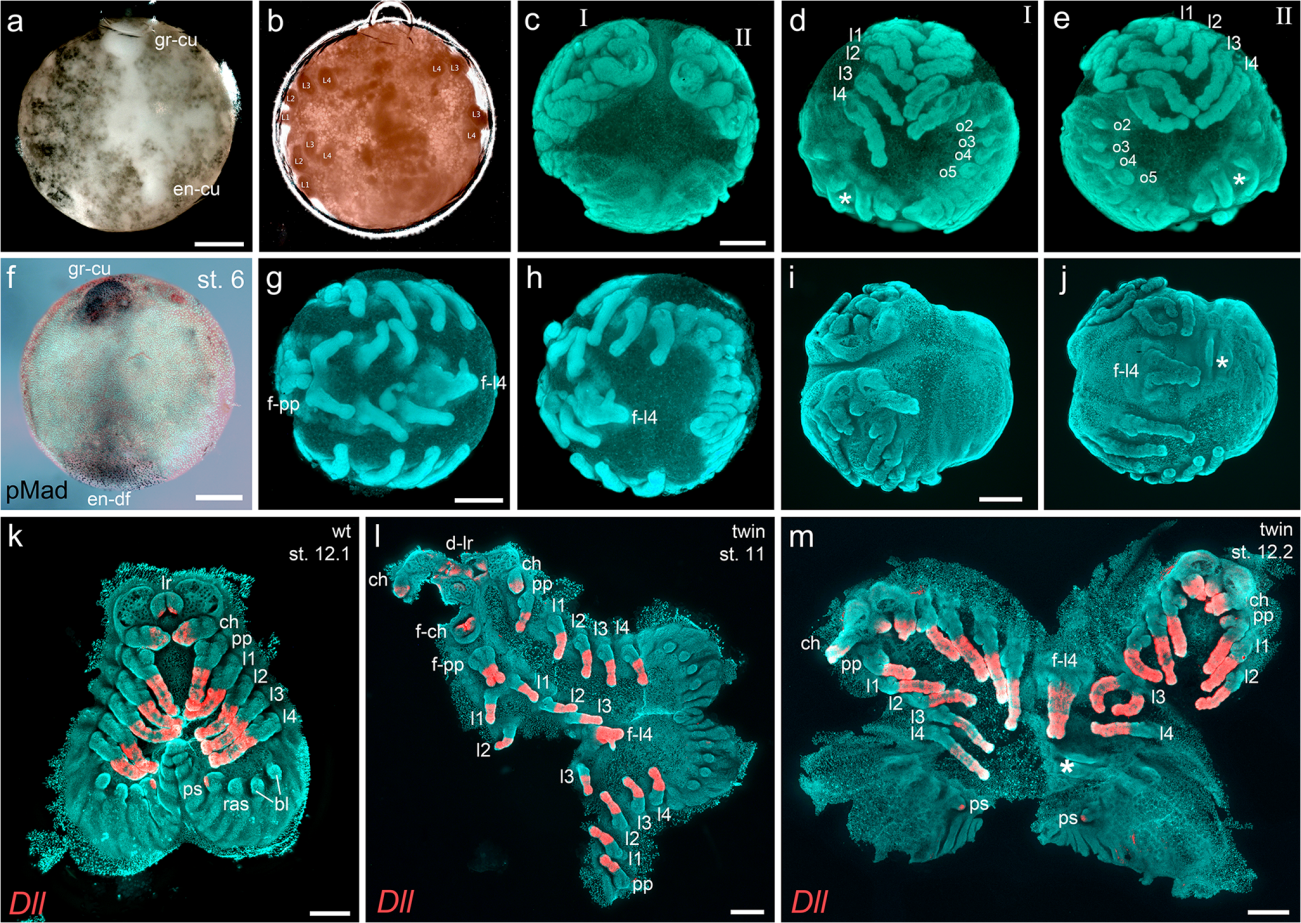
Cumulus grafting experiments in *A. geniculata.***a–e** Stills from Movie S12. **a** Bright field image of an embryo at mid stage 5. A donor cumulus has been grafted towards the opposite side of endogenous cumulus. **b** The same embryo as shown in **a** at embryonic stage 9 (transmitted light). The labels on the appendages indicate the duplication of the body axes. **c** Lateral-anterior view on the same embryo as shown in **a** and **b** at embryonic stage 12. The left and the right twin (marked by I and II) share one well-developed opisthosoma (appendage buds on o2–05 have been marked in **d** and **e**) and one malformed opisthosoma (marked by the asterisk in **d** and **e**). **d** Ventral view on twin I. **e** Ventral view on twin II. **f** pMad antibody staining marks the activated BMP signalling pathway in the region of the grafted cumulus and the endogenously developed dorsal field. **g–j** and **l,m** Two additional examples of twinning generated via cumulus grafting experiments. The embryo shown in **g** and **h** is shown as a flat mount in **l**. The embryo shown in **i** and **j** is shown as a flat mount in **m**. **k-m** flat mounted embryos stained for the appendage marker *Dll* (false-colour overlays of in situ hybridization images). In the opisthosoma, *Dll* is only expressed in the tips of the developing posterior spinnerets. For better orientation, fused pedipalps (f-pp) and walking legs (f-l4) have been marked in **g**, **h**, **j**, **l** and **m**. In addition, the asterisk in **j** and **m** marks a small duplicated opisthosoma. Abbreviations: bl, book lung; ch, chelicera; d-lr, duplicated labrum; en-df, endogenous dorsal field; en-cum, endogenous cumulus; f-ch, fused chelicera; f-l4, fused walking leg 4; f-pp, fused pedipalps; gr-cu, grafted cumulus; l1-l4, walking legs; lr, labrum; o2–5, opisthosomal segments 2–5; pp, pedipalp; ps, posterior spinneret; ras, rudimentary anterior spinneret. Scale bar is 500 μm

I performed cumulus grafting in about 50 *A. geniculata* embryos at mid to late stage 5. The ectopic cumulus was always placed within the germ-disc opposite to the endogenous cumulus, which was already migrating towards the rim of the disc (see Fig. 8a; Fig. 10b; Movie S12). The cumulus material from the donor embryo was clamped between the germ-disc and the vitelline membrane of the acceptor embryo. For technical reasons, cumuli were not transplanted below the ectoderm of the germ-disc. Nevertheless, the cumulus of the donor embryo regularly fused with the ectoderm of the germ-disc of the acceptor embryo (see Movie S12). As cumulus material from the donor embryo sometimes detached from the germ-disc of the acceptor embryo, not all analysed embryos showed an axis duplication phenotype. In addition, I failed to fix all of the cumulus-grafted embryos. In the end, I obtained 12 embryos that showed a complete or partial axis duplication phenotype. Figure 8 shows three examples of complete (Fig. 8a–e) or partial (Fig. 8g–j,l,m) axis duplication events after cumulus grafting. Embryos of the same cocoon that were not manipulated did not show any axis duplication (*n* > 100).

Some cumulus-grafted embryos were tested for ectopic BMP pathway activity. In these embryos, 15–20 h after cumulus grafting, a strong ectopic pMad signal was detectable in the region around the cumulus graft (Fig. 8f).

In his comprehensive work, Holm already showed a huge variety of axis duplication phenotypes that can occur if cumulus material is placed at different regions within the germ-disc (Holm 1952). Also in my grafting experiments, I observed many different axis duplication phenotypes. Depending on the timing, the position within the germ-disc and the amount of grafted cumulus material, twinning is more or less perfect. A kind of perfect twinning event is depicted in Movie S12 and in the schematics shown in Fig. 10b. Here, the grafted cumulus material is big enough and fuses at the right time with the germ-disc of the acceptor embryo. This leads to an ectopic dorsal-field, which is very similar in size and shape to the endogenous dorsal field. As a result, convergent extension mechanisms drive the formation of two embryos that share one posterior pole (see Fig. 10b).

In spiders, only the prosomal segments are laid down during germ-disc stage and posterior segments are added sequentially via the posteriorly located segment addition zone (reviewed in Hemmi et al. 2018; Kaufholz and Turetzek 2018; McGregor et al. 2008). A perfect twinning event leads to the formation of two opisthosomata that share the saz at early germ band stages (see stages 8–10 in Fig. 10 and the drawings of Holm (Holm 1952)). However, as a separation event between the two opisthosomata seems to occur during advanced embryonic development (see schematics in Fig. 10b and the embryo shown in Fig. 8c–e), mis-patterning of one of the duplicated opisthosomata was frequently observed (see Fig. 8d,e). In the twinned embryo shown in Movie S12 and Fig. 8a–e, it is likely that only one opisthosoma duplicate took over most portions of the saz. As a result, only one opisthosoma duplicate showed a wild type-like morphology, whereas the other duplicate was slightly deformed (marked with an asterisk in Fig. 8d,e).

Fusion of prosomal segments and imperfect twinning of embryos was probably due to the fact that grafted cumulus material was not big enough or that the fusion of the grafted material did take place at the wrong place and time. In these cases the developing embryos shared a single wild type looking opisthosoma (Fig. 8g–j,l,m; schematics in Fig. 10c).

The results show that not only in true spiders like *A. labyrinthica* (Holm 1952) and *H. adansoni* (Oda et al. 2019) but also in the basally branching tarantula *A. geniculata*, cumulus grafting is leading to an axis duplication phenotype.

### Axis duplication via local activation of the BMP signalling pathway

In spiders, the cumulus is the source of secreted Decapentaplegic protein (Dpp; homologous to the vertebrate BMP2/4 protein), and the knockdown of *dpp* or the loss of the cumulus results in embryos that fail to break the radial symmetry of the germ-disc (Akiyama-Oda and Oda 2003; Akiyama-Oda and Oda 2006; Pechmann et al. 2017).

**Fig. 9:**
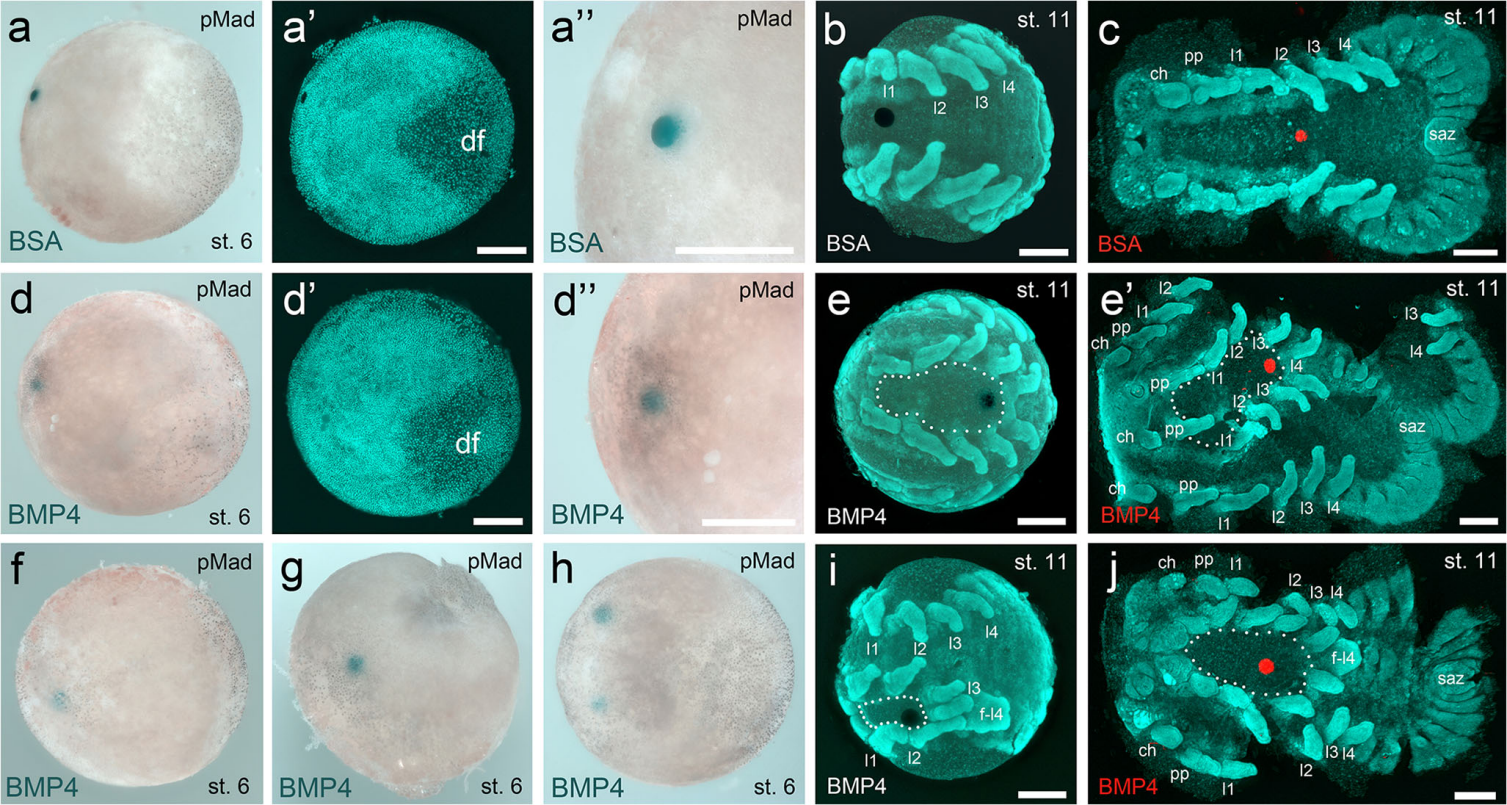
Axis duplication via local BMP pathway activation. **a,a’** A BSA-soaked agarose bead is not able to locally activate the BMP signalling pathway. pMad signal is only visible in the region of the dorsal field (df). **a”** Magnified view on the region of the transplanted bead. **b,c** Embryos develop normal and show no axis duplication. **d,d’** An agarose bead soaked with human BMP4 is able to locally induce the BMP signalling pathway. **d”** Magnified view on the region of the transplanted bead. **f–h** Three embryos in which a single BMP4-soaked bead (**f**,**g**) or multiple BMP4-soaked beads (**h**) have been transplanted. Nuclear pMad signal is detectable in a broad region around the transplanted beads. **e,e’,i,j** In the shown embryos, a BMP4-soaked bead was transplanted to the germ-disc (opposite to the cumulus). The local activation of the BMP signalling pathway did lead to axes duplication phenotypes during further embryonic development. **a**,**a”**,**d**,**d”**,**f–h** Bright field (the agarose bead is visible as a blue spot). **a’**,**b**,**e**,**i** Nuclear Sytox green staining (the agarose bead is visible as a black spot). **c**,**e’**,**j** Nuclear Sytox green staining (false colour overlay of the agarose bead; visible as a red spot). **c**,**e’**,**j** Flat mounted embryos. Same embryo in **e** (whole mount) and **e’** (flat mount). Abbreviations: ch, chelicera; df, dorsal field; f-l4, fused l4; l1-l4, walking legs; pp, pedipalp; saz, segment addition zone. Scale bar is 500 μm

Regarding the functional analysis of the cumulus, only loss of function experiments has been performed so far, and the final proof that Dpp is sufficient to induce the establishment of the dorsal field is still missing. It has been shown that arthropod Dpp is able to activate the BMP signalling pathway in vertebrate species and vice versa (Padgett et al. 1993; Sampath et al. 1993). For this reason, I have transplanted human BMP4-soaked agarose beads to the rim of the germ-disc of stage 5 *A. geniculata* spider embryos. To better mimic the function of the endogenous cumulus, beads were transplanted below the ectoderm of the disc. As a control, BSA-soaked beads were transplanted to embryos of the same cocoon. Embryos were fixed 15–20 h after bead transplantation, and the activation of the BMP signalling pathway was analysed via pMad antibody staining. In 100% of the control embryos, no pMad signal was detectable around the transplanted bead (*n* = 29; see Table 1; Fig. 9a–a”). In contrast, the transplantation of BMP4-soaked beads led to a detectable pMad signal in 50% of the analysed embryos (*n* = 32; see Table 1; Fig. 9d–d”,f–h). This result shows that human BMP4 is able to locally induce the BMP signalling pathway in the germ-disc of *A. geniculata* germ-disc stage embryos. However, the direct comparison between cumulus-grafted and BMP4 bead-transplanted embryos showed that an ectopic cumulus graft (a strong pMad signal around the cumulus graft was visible in 90% of the embryos) is more reliably inducing the BMP signalling pathway in manipulated embryos (see Table 1).

**Table 1:** Analysis of the activated BMP signalling pathway in cumulus-grafted and bead-transplanted embryos

	pMad signal around the endogenous cumulus/in the endogenously induced dorsal field	pMad signal around the grafted cumulus /in the ectopically induced dorsal field	pMad signal around the transplanted bead	number of analysed embryos
embryos with ectopic cumulus graft	10	9	n/a	10
BSA bead-transplanted embryos	29	n/a	0	29
BMP4 bead-transplanted embryos	32	n/a	16	32

Embryos were analysed for nuclear localized pMad 15–20 h after cumulus grafting or bead transplantation, respectively. Please note, only embryos in which the ectopic cumulus graft fused to the germ-disc were used for the analysis

Nevertheless, although a pMad signal in late stage 5/early stage 6 embryos was only detectable in 50% of the BMP4 bead-transplanted embryos, 85% of the transplanted embryos did show an axis duplication phenotype during later stages of development (*n* = 40; e.g., see Fig. 9e,e’,i–j). The remaining 15% of the BMP4 bead-transplanted embryos showed a wild type or an unspecific phenotype (not shown). This indicates that the BMP signalling pathway around the transplanted bead was only slightly activated (below the detection level via pMad antibody staining) in a large portion of the embryos. Control, BSA-soaked beads (e.g., Fig. 9b,c) did never induce an axis duplication phenotype (*n* = 38; 36 embryos were wild type-like; 2 embryos showed an unspecific phenotype).

**Fig. 10:**
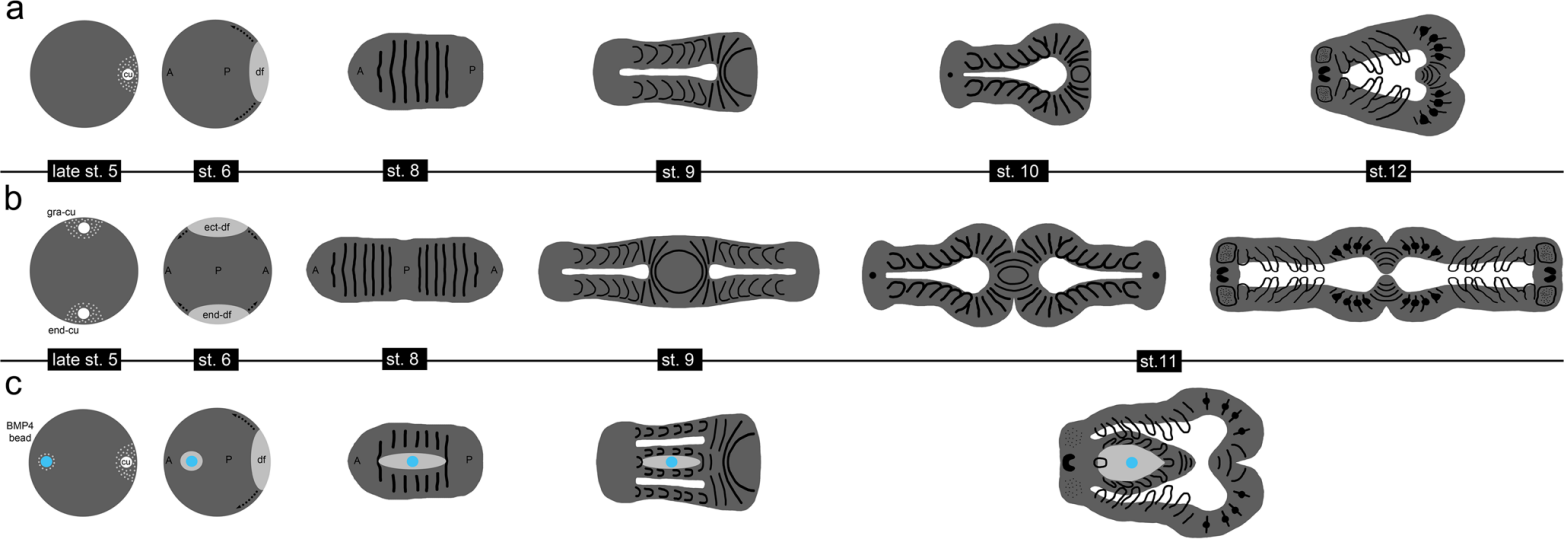
Schematics of axes duplication in *A. geniculata* embryos. **a** Wild type development. At the end of stage 5, the cumulus (cu) has shifted to the rim of the germ-disc and is inducing the dorsal-field (df). Extensive convergent extension mechanisms (indicated by the arrows at stage 6) are leading to the axially symmetric spider embryo. Segmentation is initiated and appendages grow out from the germ-band (st. 8 and 9). The ventral sulcus (white area in st. 9–12) appears and broadens. **b** Indicated is the hypothetical situation of an ideal twinning event. As the complete rim of the germ-disc has the potential to become the anterior of the embryo, a grafted cumulus (gra-cu) is able to induce a secondary body axis. Convergent extension mechanisms (indicated by the arrows at stage 6) are leading to a double-headed embryo that shares the posterior pole. Somewhere between stage 10 and stage 12, a separation event of the segment addition zone occurs (compare to the embryo shown in Fig. 8a–e and Movie S12). **c** BMP4 bead (indicated in blue) transplantation often resulted in the induction of a small ectopic dorsal-field (grey area surrounding the blue bead in st. 6–11) in the prosoma of the developing embryo. This results in partial axis duplication within the prosoma of the germ-band. At the anterior and posterior region of the ectopically induced area the event of appendage fusion might take place. All embryos have been drawn as flat mounts. Please note that in **a,b** the ectopic or endogenous dorsal-field is not indicated from stage 8 onwards. In **a** and **c** anterior (A) is towards the left. In **a** and **c**, posterior (P) is in the centre (stages 5 and 6) or towards the right (germ-band stage). In **b**, as a result of the axis duplication, posterior is always in the centre. Activated BMP signalling pathway is indicated via the grey dots (see late stage 5 embryos)

In contrast to the cumulus transplantation (see Fig. 8a–e), I never observed a “perfect” axis duplication phenotype upon BMP4 bead transplantation. Most embryos showed an axis duplication event within the prosoma of the embryo (Fig. 9e,e’,i–j). As a result, fusion of prosomal appendages was frequently observed (Fig. 9e, e’,i–j; Fig. 10c). One of the strongest phenotypes upon BMP4 bead transplantation is shown in Movie S13. In this embryo almost the entire prosoma was duplicated and only the central part of the fourth walking leg segment was fused.

Overall, these results are the final proof that the local activation of the BMP signalling pathway within the germ-disc is sufficient to induce a secondary body axis in spider embryos.

## Conclusions and future directions

*A. geniculata* is a promising model system that can be used to answer a variety of evolutionary developmental questions. It can be reared in the lab throughout the year, genomic and transcriptomic sources are available (this work; Sanggaard et al. 2014), and the embryos are amenable to standard molecular biological techniques like in situ hybridisation and antibody staining (this work; Pechmann and Prpic 2009). Future studies will show if it is possible to use embryonic RNA interference to analyse gene functions. The observation that RNA probes are cross hybridising across tarantula genera (Fig. S7) might help to compare gene expression pattern across mygalomorph species.

It will be interesting to see whether anterior as well as posterior segmentation mechanisms are conserved between mygalomorph and araneomorph spider species. Also the timing of the establishment of the cumulus seems to be different between mygalomorph and many araneomorph spider species, and it will be interesting to see whether the same set of genes is expressed with in the cumuli of distantly related spiders.

In this study I have demonstrated that *A. geniculata* embryos are well suited to perform grafting and transplantation experiments. In future studies, especially the bead transplantation experiments will allow analysing the local activation of different signalling pathways at different regions within the developing spider embryo.

## Electronic supplementary material

Movie S1 Early developmental stages of *A. geniculata* (side view; stages 1–6). The movie starts at mid stage 1 of embryonic development. As the two embryos swim directly below the surface of the oil, a reflection of the embryo is visible in the upper part of the frame. Polygonal cortical fields are already established. Energids reach the polygonal cortical fields at 1400 min. At the same time, energids fuse with most of the cortical fields and contraction of the embryo is initiated. Please note that energids reach the surface all around the egg (compare to Movie S2). Contraction is the beginning of stage 2. The process of embryo contraction is completed at 2700 min. Energid free cortex fields contract between minute 9000 and 11000 (compare to Fig. S4). The result is a regular blastoderm that is interspersed with white spots. This is stage 3 of embryonic development. At stage 4, the primary thickening is established (not visible in this side view, see Fig. 3c and Movie S7). The cumulus appears at stage 5 (appears in the left embryo at 12700 min; right embryo at 13200 min) and shifts towards the rim of the germ-disc (takes until 14000 min in the left embryo and until 15000 min in the right embryo). As soon as the cumulus starts to migrate, gastrulation is initiated. The majority of the gastrulating cells originate from the central primary thickening. However, smaller gastrulation events also occur at multiple positions within the embryo (e.g., Movie S6). The cumulus disappears at stage 6, and the cumulus cells seem to spread over the extra-embryonic area. (MP4 23,751 kb)

Movie S2 Contraction process in *B. albopilosum* (side view). The movie starts at early stage 1 of embryonic development. Polygonal cortical fields are about being established. The upper third of the egg appears more transparent. The cleavage energids reach the surface of the egg only in the lower two third of the egg (in the region of the densely packed yolk granules; 4000 min). The upper, energid-free cortex, collapses/contracts on top of energids that have arrested at the surface of the densely packed yolk mass. Contraction is completed at minute 5700. The video ends at early stage 2 of embryonic development. (MP4 23,014 kb)

Movie S3 Establishment of polygonal cortex fields. Magnified view of a central region of Movie S2. An internal contraction (probably due to a round of energid cleavage) occurs at the time when the polygonal cortical fields are forming. (MP4 3629 kb)

Movie S4 Contraction process in *B. albopilosum* (top view). Transmitted light conditions. Within each cortical field, attachment points of the strands that connect the energids to the cortex are visible as small black spots. Some cortical fields have been damaged. This allows imaging the strands that connect the energids with the cortex (see magnified view in Movie S5). (MP4 22,916 kb)

Movie S5 Energid/cortex connecting strands. Magnified view of the central region of Movie S4. The movie loops two times. Compare to Fig. S3e,e’. (MP4 7369 kb)

Movie S6 Early determination of different cell types. Magnification of the central part of the left embryo shown in Movie S1 (at 11600 min). Some cells appear whitish (marked in red). These cells become migratory when gastrulation starts at stage 5 (compare to Movie S1). (MP4 7222 kb)

Movie S7 Embryonic development of *A. geniculata* (top view; stages 2–14). 16.5 days of constant live imaging. Movie starts at late stage 2. A regular blastoderm is established at stage 3 (around 3000 min). Cells aggregate and form the primary thickening (stage 4; aggregation process is completed at 4800 min). The cumulus appears at stage 5 and gastrulation is initiated. The primary thickening is the source of most gastrulating cells. Gastrulating cells spread over the germ-disc, and the cumulus shifts towards the rim of the disc. The cumulus disappears at stage 6 (around 7000 min), and the dorsal field is established (compare to Fig. 3f). Stage 6 is between minute 7000–7680 and stage 7 until minute 8300. The axially symmetric stage 8 embryo is formed. Appendage buds appear in the prosomal segments. Embryonic stages 9–13 (minutes 9500–17500; compare to stages shown in Figs. 5, 6, and 7). First signs of egg tooth pigmentation are visible around minute 17500. Pigmentation of the false pincer of the chelicera and bristles starts at minute 20940. Muscle twitching starts around minute 22000. (MP4 23,532 kb)

Movie S8 Embryonic development of *B. albopilosum* (top view; stages 2–12). Transmitted light conditions. Stage 2 is until 3200 min. Stage 4 is at around 4500 min. Cumulus migration (stage 5) is from minute 5000–6500. Stage 8 is at around 9500 min. The movie ends at embryonic stage 12. (MP4 23,357 kb)

Movie S9 Embryonic development of *A. geniculata* (side view; stages 3–11). The movie starts with the formation of the primary thickening. Cumulus migration is difficult to observe. In both embryos, the cumulus migrates towards the right. The cumuli disappear at around 1700 min. At minute 3000, embryonic stage 8 is completed. Outgrowth of the appendages is visible until the end of the movie. Anterior is to the left. (MP4 23,646 kb)

Movie S10 Embryonic development of *A. geniculata* (top view; stages 9-hatching). Embryonic stages 9–13 (minutes 0–7900; compare to stages shown in Figs. 4, 5, and 6). Egg tooth pigmentation starts around minute 7900. Pigmentation of the false pincer of the chelicera and bristles starts around minute 11500. The postembryo hatches from the egg at the end of the movie. (MP4 23,382 kb)

Movie S11 The postembryo hatches from the egg. Shortly before hatching, the embryo turns inside the egg. This might lead to perforation of the egg membrane via the egg tooth. Hatching takes around 30 min (minutes 300–330). Subsequently, the postembryo is heavily moving its prosomal appendages. (MP4 23,907 kb)

Movie S12 Axis duplication after cumulus grafting. A cumulus from a stage 5 donor embryo has been grafted towards the opposite side of the migrated endogenous cumulus of the acceptor embryo. Both cumuli disappear at around the same time (10 h after transplantation). The grafted cumulus induces an ectopic dorsal field. This is resulting in the development of a twinned embryo. After 70 h a transmitted light image has been taken to better illustrate the development of the appendages. Posterior of the embryo is in the centre of the movie. The left and the right twin have grown around the yolk, and only some walking legs of the prosoma are visible (labelled L1-L4 in the left twin and L3-L4 in the right twin). The twinned embryo has been fixed at stage 12 of development. The fixed embryo has been stained with the nuclear dye Sytox green. A lateral view and a ventral view on the left and the right twin are shown at the end of the movie. For a better understanding of the twinning process, please compare to the schematics shown in Fig. 10b. (MP4 21,952 kb)

Movie S13 Axis duplication after BMP4 bead transplantation. A BMP4-soaked agarose bead was transplanted towards the germ-disc (opposite to the endogenous cumulus). The bead induced axis duplication in the head and prosoma of the spider embryo. The embryo was fixed at stage 9 of development. The fixed embryo has been stained with the nuclear dye Sytox green. A lateral, anterior and a posterior view of the fixed embryo are shown at the end of the movie. Abbreviations: ch, chelicera; L1-L4, walking legs; pp., pedipalp; saz, segment addition zone. (MP4 24,259 kb)

ESM14 (PDF 81597 kb)
