# Supplementary material for: Embryonic development and secondary axis induction in the Brazilian white knee tarantula Acanthoscurria geniculata, C. L. Koch, 1841 (Araneae; Mygalomorphae; Theraphosidae)
Source: Dev Genes Evol. 2020 Feb 19;230(2):75–94. doi: 10.1007/s00427-020-00653-w (PMC7128004; doi:10.1007/s00427-020-00653-w)
Supplement: Supplementary file 14 — (PDF 81597 kb) [file 427_2020_653_MOESM14_ESM.pdf]

Supplementary Material

Embryonic development and secondary axis induction in the Brazilian white knee tarantula *Acanthoscurria geniculata*, C. L. Koch, 1841 (Araneae; Mygalomorphae; Theraphosidae).

Matthias Pechmann
Institute for Zoology, Department for Developmental Biology, Biocenter,
University of Cologne, Zuelpicher Str. 47b, 50674 Cologne, Germany

Table S1: Primer List

Primer name	Primer Sequence	TRINITY_ID
Ag-cad-fw	5′- GTG TAT ACC GTG CCT AAC GG -3′	TRINITY_DN73996
Ag-cad-rev	5′- CTT GGA ACA ACA CTG TAA GTG G -3′	
Ag-fascin-fw	5′- CTC AAG AGT CAT CTC AAC AAG TAC C -3′	TRINITY_DN83029
Ag-fascin-rev	5′- CAG ATA TAT GCT TTG ATA CGT AGC C -3′	
Ag-Dll-fw	5′- GTA TGG CGG GTA CTG CGG ATG -3′	TRINITY_DN74055
Ag-Dll-rev	5′- GAT TAA GTG AGA ATT TGT TGG CTC ATT G -3′	
Ag-en-fw	5′- CAA GAG AAT CGG TAC CTG AC -3′	TRINITY_DN81039
Ag-en-rev	5′- GCT GTT TGT ACA CAC CAA AGT AC -3′	
Ag-eve-fw	5′- GTT ATT ACA GCA GGA GGA AAT GAG -3′	TRINITY_DN74153
Ag-eve-rev	5′- CAG CAT TAG TCC CAG CTA TGA AG -3′	
Ag-hairy-fw	5′- CTC TCT ACT CGA TCG GAT CTA AC -3′	TRINITY_DN68592
Ag-hairy-rev	5′- GTC CAT GTG TTT GTT TAC TAT GTA CAG -3′	
Ag-otd-fw	5′- CAA TAT GAC TAT GGA GAT TCT GAG C -3′	TRINITY_DN82202
Ag-otd-rev	5′- GGC TGA ACT CCT ATC ACC GG -3′	

Table S2: Basic statistics for the Trinity assembly and BUSCO analysis

	Ag_Pechmann_Trinity Assembly
Number of contigs	242.018
Total length (nt)	159.990.564
Longest contig (nt)	20.105
Shortest contig (nt)	201
Mean contig length (nt)	661
Median contig length (nt)	346
N50 contig length (nt)	1.081
Number of contigs in N50:	35.755
GC Content of contigs:	37,91%
Metazoa BUSCOv2/3	C:98.5%[S:58.4%,D:40.1%],F:1.1%,M:0.4%
Arthropod BUSCOv2/3	C:98.1%[S:56.9%,D:41.2%],F:1.4%,M:0.5%

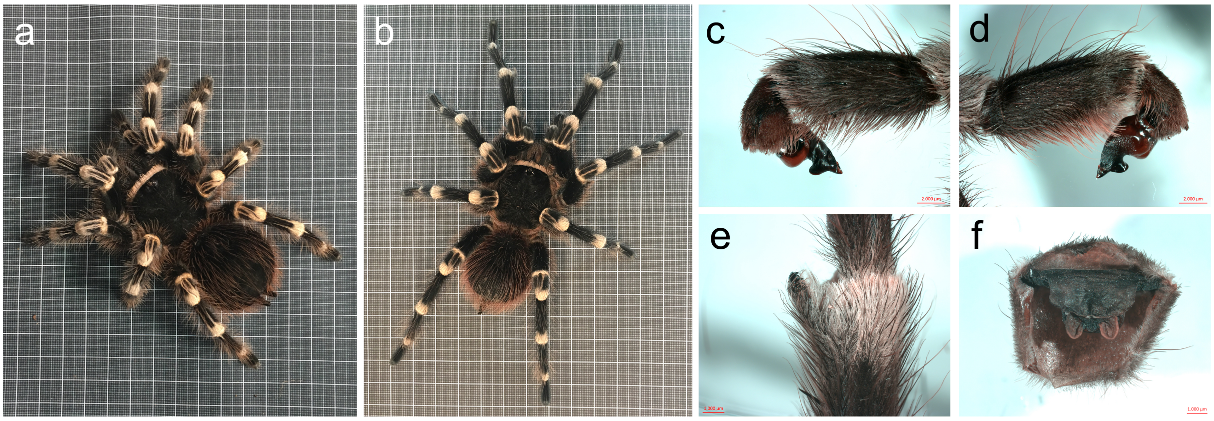

Fig. S1 Sex specific characters of *A. geniculata*. a An adult female on scaling paper. **b** An adult male on scaling paper. **c-d** Left and right view on the copulatory organ (bulb) of an adult male. **e** The tibial apophysis of on the first walking leg of an adult male. **f** The receptaculum semenis an adult female.

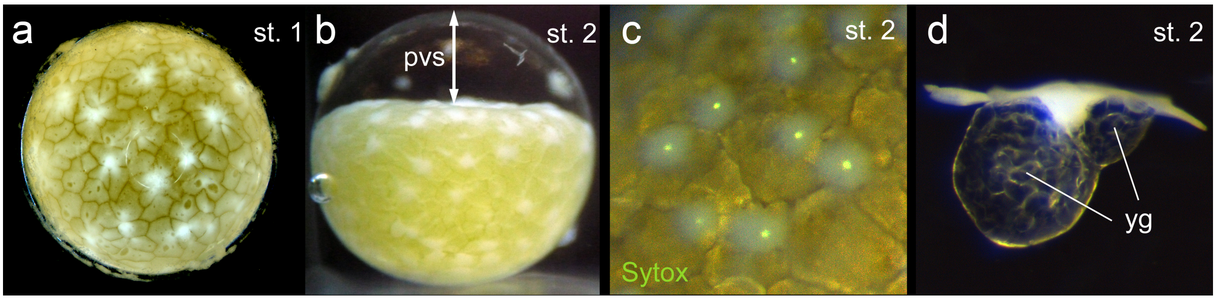

Fig. S2 Early developmental stages of tarantula embryos. a The energids invade the cortex of the egg. Each energid fuses with several polygonal cortical fields. **b** Side view on an early stage 2 embryo. The perivitelline space (pvs) is visible. **c** A close up on the surface of a fixed stage 2 embryo stained with the nuclear dye Sytox green. **d** A single cell of a fixed stage 2 embryo has been isolated. Side view. The central part of the cell containing the nucleus extends deep between the big yolk granules (yg).

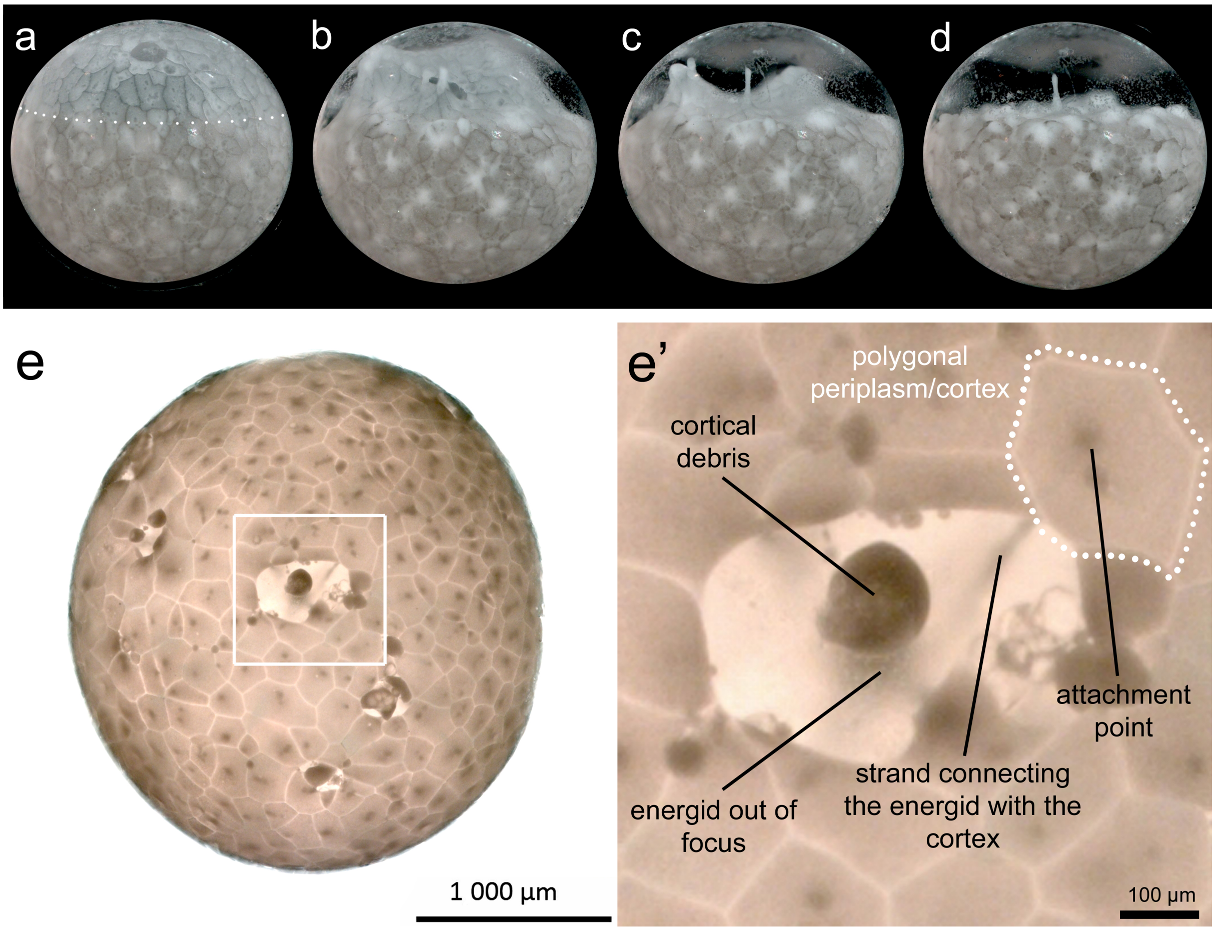

Fig. S3 Condensation process in *B. albopilosum*. a-d Stills from Movie S2. **e** Still from Movie S4. **a** The upper third of the egg (marked by the dotted line) appears more transparent. The cleavage energids reach the surface of the egg only in the lower two third of the egg (in the region of the densely packed yolk granules). **b-d** The upper, energid free cortex, collapses on top of energids that have arrested at the surface of the densely packed yolk mass. **e-e'** While handling the eggs, some polygonal cortical fields got damaged (cortical debris). This allows imaging the strands that connect the energids with the cortex (compare to Movie S4 and S5).

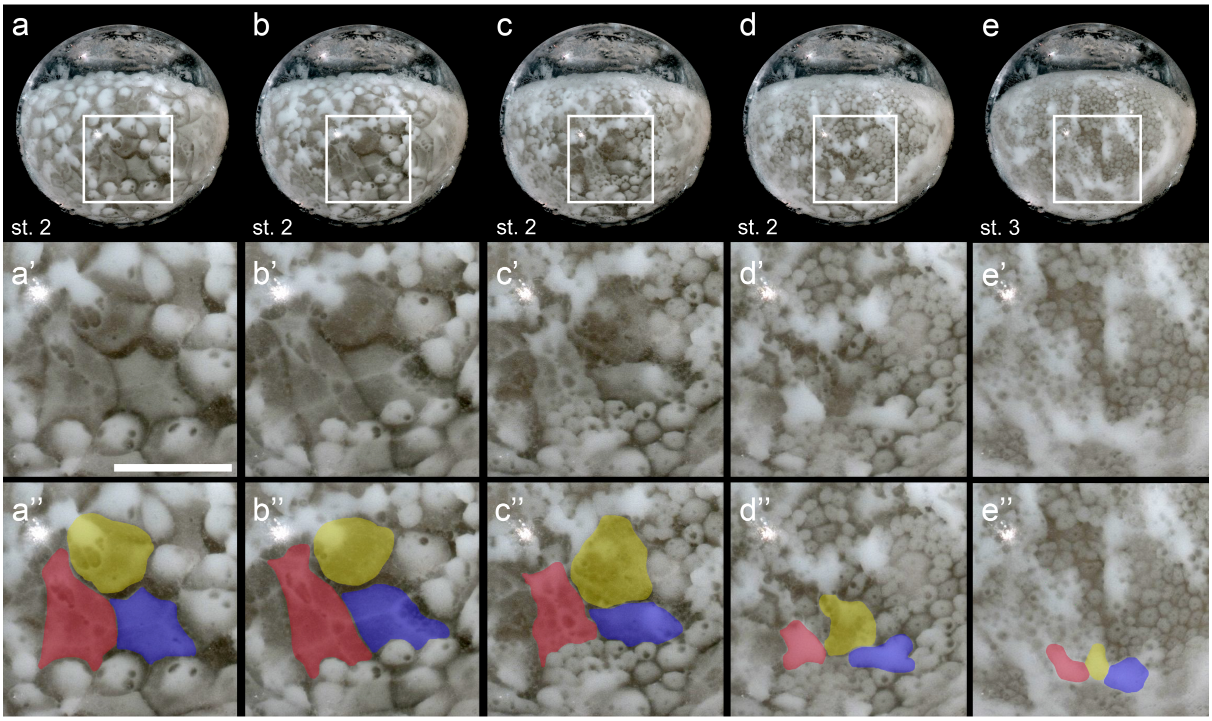

Fig. S4 The development of white spots of stage 3 embryos. Stills from Movie S1. Boxed regions in **a-e** have been magnified in **a'-e'**. In **a''-e''**, energid free polygonal cortex fields are coloured in red and blue. A cortical field containing a small energid is marked in yellow. The big polygonal cortical fields contract during stage 2 and are leading to most of the white spots that are visible at stage 3 of embryonic development. Scale bar is 500 µm.

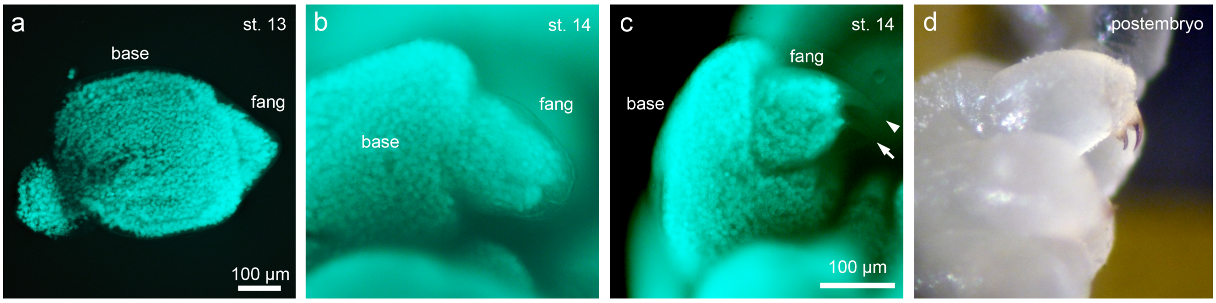

Fig. S5 Development of the chelicera. a At embryonic stage 13, the chelicera is divided into the proximal base and the distal fang. **b-c** During stage 14, a dorsally (arrowhead in c) and ventrally (arrow in c) located tooth is secreted (the so called "false pincer" (Galiano 1996)) from the distal tip of the fang. **d** These teeth are strongly sclerotized at the postembryonic stage (compare to Fig. S6, Movie S7 and S10).

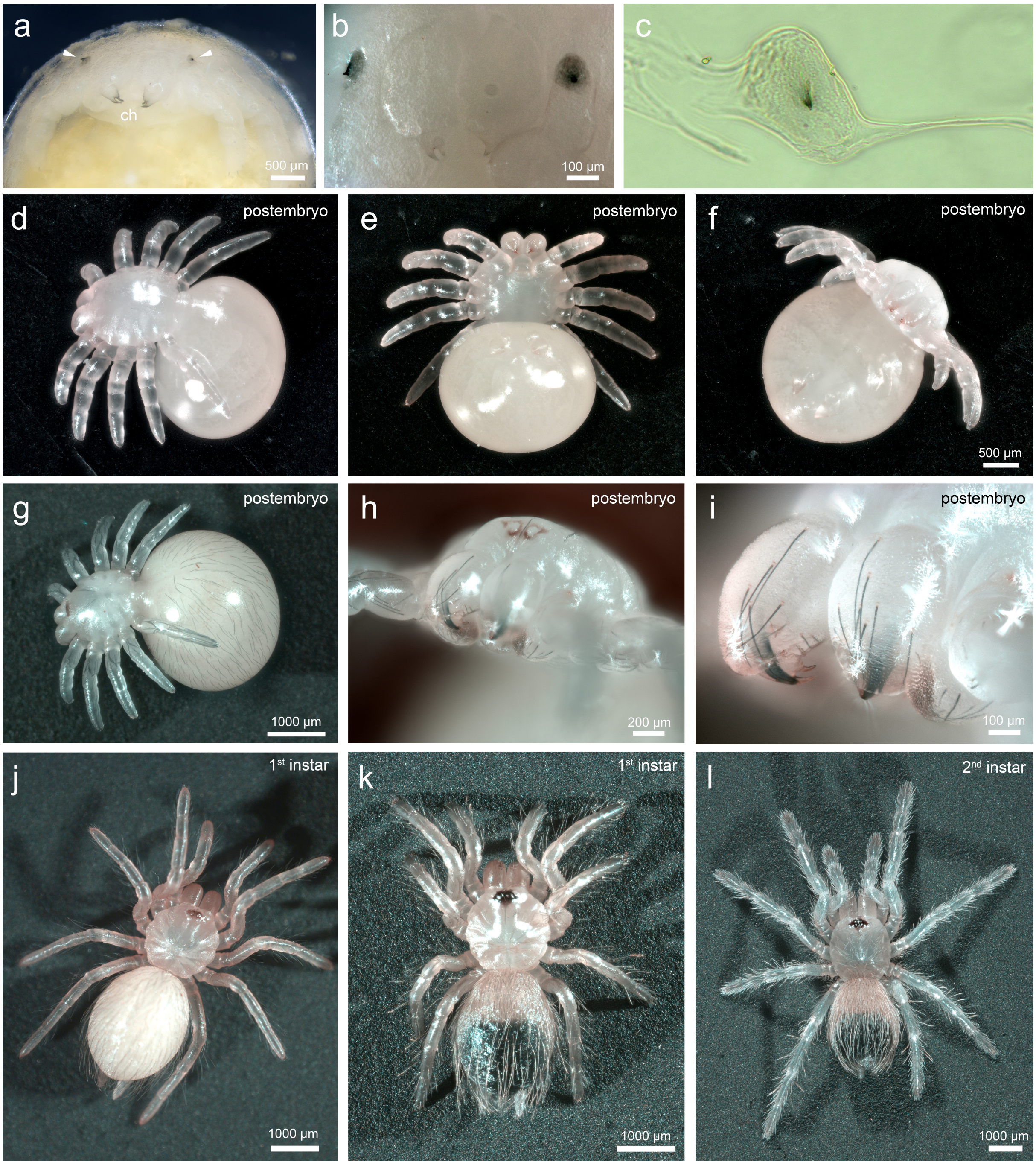

Fig. S6 Postembryonic development of *A. geniculata*. a, b Postembryo shortly before hatching. The egg tooth (arrowheads in **a**, black structures in **b**) and the false pincer of the chelicera (ch) are strongly sclerotized. **c** Phase contrast image of the egg tooth. The egg tooth is a small structure that is only loosely connected to the cuticle of the postembryo and often detaches during the hatching process. **d-i** The postembryo is commonly known as "eggs with legs". At the beginning, the postembryo is shiny and mostly unpigmented (**d-f**) but is able to move its legs (see MovieS11). **g-h** After a few days the new cuticle develops under the postembryonic cuticle. Pigmentation of hairs and eyes is visible through the postembryonic cuticle. **i** The fang of the chelicera has developed and projects into the dorsal tooth of the false pincer. **j and k** First instar larva. Depending on the temperature, this stage can take several weeks. Internal organs like silk glands are developing. After several days, first instar larva are able to produce silk (not shown). **k** First instar larva shortly before moulting into the second instar larva. A field of urticating hairs is developing on the opisthosoma of the new cuticle. This field is visible as a dark patch and is shining through the cuticle of the first instar larva. **l** The second instar is able to actively hunt its pray.

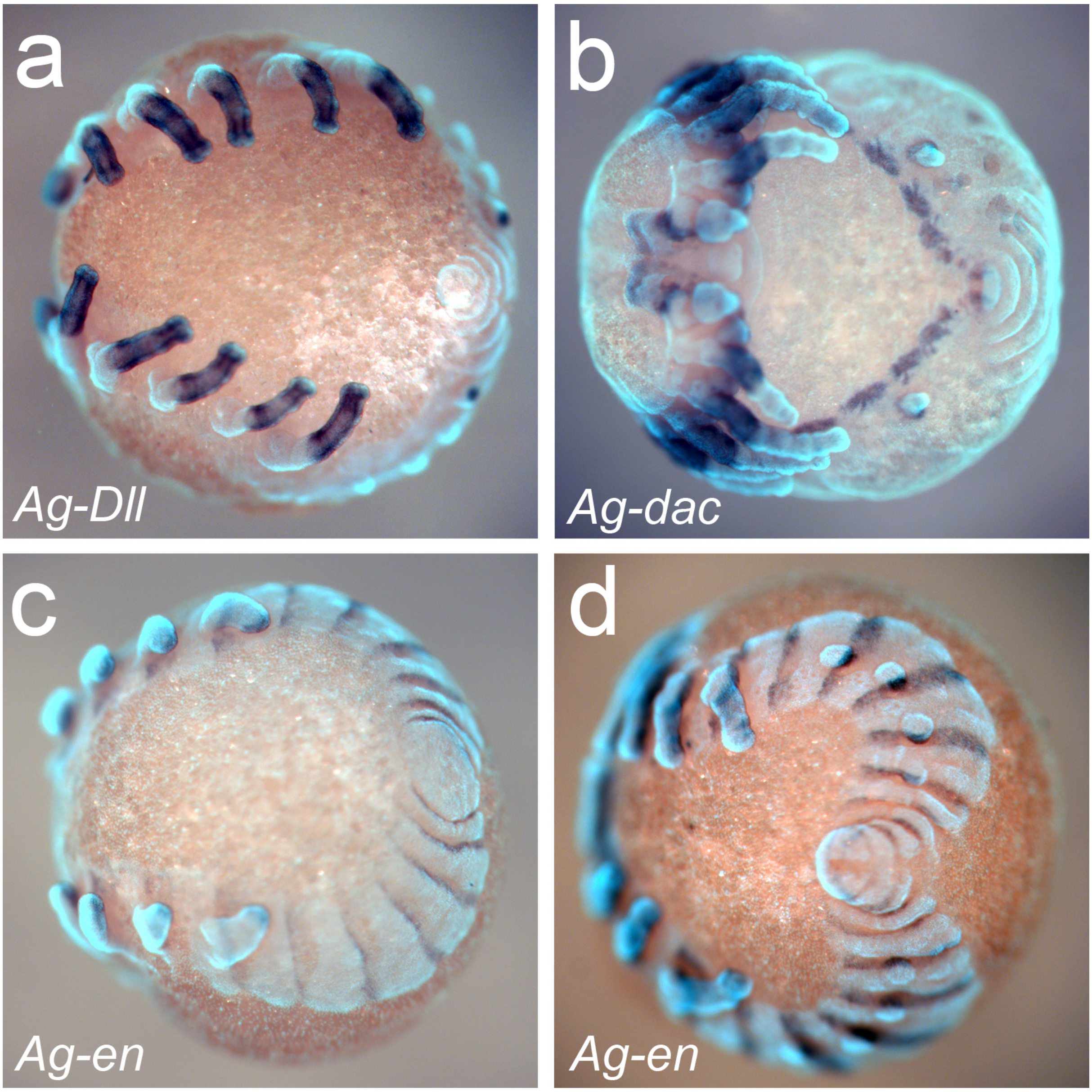

Fig. S7 Cross-hybridising probes in *B. albopilosum*. **a-d** *In situ* hybridisation in *B. albopilosum* embryos using *A. geniculata* probes for the genes *Dll*, *dac* and *en*.

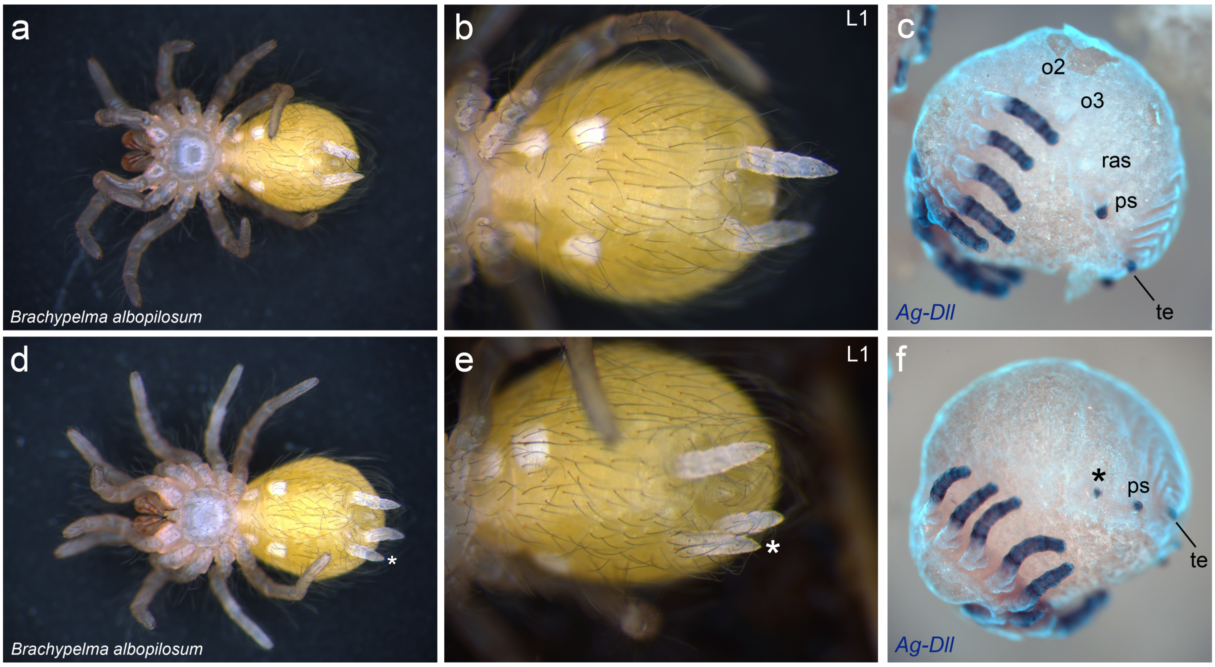

Fig. S8 Spontaneous reactivation of the rudimentary anterior spinneret. a, b, d, e Ventral views on the first instar larva of *B. albopilosum***.** Some spiders spontaneously developed a third spinneret (asterisk in **e**) that was morphological similar to the regular posterior spinnerets. **c** and **f** The cross-hybridizing *Ag-Dll* probe (see Fig. S7a) was used to visualize the expression of *Dll* in *B. albopilosum* embryos. **c** In wt, *Dll* is expressed in the prosomal appendages, the telson (te) and in the posterior spinneret (ps). In wild-type embryos, *Dll* is not expressed in the rudimentary anterior spinneret (ras), which degenerates over time. **f** Some embryos showed a reactivation of *Dll* expression in the appendage buds of the 4th opisthosomal segment (see asterisk in **f**). These embryos presumably developed into spiders that showed an ectopic spinneret.
